# From eco-consciousness to apathy: the ECO-SHADOW inventory to assess cognitive and behavioral affect regulation and its role in climate action

**DOI:** 10.3389/fpsyg.2025.1575185

**Published:** 2025-09-09

**Authors:** Monika Lohani, Wei Wei, Benjamin Janney, Lynne Zummo, Ginger R. Blodgett

**Affiliations:** ^1^Applied Cognition Lab, Department of Psychology, University of Utah, Salt Lake, UT, United States; ^2^Natural History Museum of Utah, University of Utah, Salt Lake, UT, United States; ^3^Department of Educational Psychology, University of Utah, Salt Lake, UT, United States

**Keywords:** climate change, cognition and behavior, affect regulation, psychometrics, wellbeing, sustainability

## Abstract

The climate change crisis continues to have interrelated health, economic, and societal consequences; yet how people psychologically manage these challenges remains underexplored. Specifically, people may have distinct ways of dealing with the realities of climate change, which can impact their wellbeing and influence their engagement in climate action. Thus, the current work aimed to evaluate how people manage their cognition and behavior specific to climate change. We developed and validated a new comprehensive measure called ECO-SHADOW to assess regulated responses to climate change. The existing literature on climate change was integrated with theoretical perspectives from affect regulation literature to generate potential strategies for managing cognitions and behaviors. Based on data collected (*N* = 566), exploratory factor analysis identified nine affect regulation factors underlying nearly 60 strategies: Eco-consciousness, Conflict, Outcast, Spirituality, Hope, Apathy, Doom, Overplay, and Withdrawal. The ECO-SHADOW inventory is a reliable, valid, and currently the most exhaustive measure of the wide-ranging cognitive and behavioral regulation strategies employed to process climate change challenges, with some being more adaptive toward climate action (such as eco-consciousness and hope), while others being maladaptive (including apathy, withdrawal, doom, or overplay). Further work is needed to examine how affect regulation efforts relate to addressing the climate change crisis. We hope that the ECO-SHADOW inventory inspires future research promoting effective affect regulation and its connections to sustainable climate action.

## 1 Introduction

Given that the urgency and seriousness of climate change are widely known, how people manage their affective responses to environmental challenges has implications for their personal wellbeing and their engagement in sustainability efforts to mitigate climate change (Homburg et al., 2007; Ojala, 2013; Reser and Swim, 2011; Stokols et al., 2009). Still, a limited understanding exists regarding the diverse ways people regulate their emotional reactions to the climate crisis. To address this knowledge gap, we developed and validated a comprehensive measure of the regulatory approaches people may adopt to manage their responses to climate change. Understanding the regulatory strategies matters because it can help promote effective ways of dealing with the climate change crisis, support mental health, and facilitate climate action. In this paper, we first provide the background on the climate change crisis that informed this work, including the impact of climate change on emotion and wellbeing, theoretical perspectives on managing climate change reactions, and specific regulatory efforts that potentially influence responses to climate change.

As climate change progresses, the number of corresponding environmental stressors will increase, negatively impacting daily wellbeing (Thornton et al., 2014; Lohani et al., 2025c). Given the uncertainty associated with climate change, it follows that people will exhibit varying emotional responses (Pihkala, 2022a). In fact, research has reliably shown that individuals can have different outlooks and attitudes toward climate change, with some being alarmed while others are dismissive of the climate crisis (Chryst et al., 2018; Hine et al., 2016; Leiserowitz et al., 2013; Metag et al., 2017). This wide-ranging variability in climate change outlook is inherently linked to their emotional responses. Furthermore, recent work demonstrating the links between climate change-related emotions, health, and motivation highlights the importance of understanding the different emotional reactions to climate change (Böhm et al., 2023; Salas Reyes et al., 2021; Verlie, 2019). Dysregulated negative climate change emotions have been correlated with insomnia symptoms and worse mental health across multiple countries (Ogunbode et al., 2021). At the same time, experiences of negative emotions, especially at a manageable level, can be useful in promoting sustainability efforts (Wong-Parodi and Feygina, 2021). Furthermore, some negative climate change emotions (such as fear) are also linked to increased intention to engage in climate activism and pro-environmental behavior (Davidson and Kecinski, 2021; Skurka et al., 2018). These findings suggest that emotions are critical to how we mentally adapt to and act on climate change challenges (Brosch and Steg, 2021; Lohani et al., n.d.-b; Lohani et al., n.d.-a; Ogunbode et al., 2019). However, it is essential not only to understand the range of emotions related to climate change but also to recognize the diversity of ways we regulate those emotions and the consequences of those regulatory strategies (Lohani et al., 2025b; Lohani et al., 2025a; Lohani et al., n.d.-b; Lohani et al., n.d.-a). Next, we discuss the theoretical perspectives on dealing with climate change reactions.

### 1.1 Theoretical perspectives informing the regulation of climate change reactions

*Affect regulation* encompasses all attempts to influence which emotions one has and how they are experienced and expressed (Gross, 1998). The approaches people adopt to manage their emotions are called *affect regulation strategies* (Parkinson and Totterdell, 1999). People can adopt a range of affect regulation strategies to manage their emotional responses in daily life (Heiy and Cheavens, 2014). Classic work on the taxonomy of affect regulation approaches has explored ways to categorize affect regulation strategies (e.g., Aldao et al., 2010; Larsen, 2000; Lazarus and Folkman, 1984; Naragon-Gainey et al., 2017; Parkinson and Totterdell, 1999). One of the most common dimensions used for categorizing affective strategies is to distinguish between cognitive (“ways to think about it”) and behavioral (“ways to do something about it”) approaches (e.g., Larsen, 2000; Parkinson and Totterdell, 1999). Given the relevance of both cognitive and behavioral approaches, the current study examined them to understand how people manage their responses to climate change. While there may be hundreds of strategies in general, some affect regulation strategies are more relevant than others in the specific context of climate change. Therefore, we examined the affect regulation strategies most relevant to climate change. Furthermore, meta-analytic reviews and other empirical research have identified a connection between the use of specific strategies and mental health, leading to two broad categories—adaptive versus maladaptive (e.g., Aldao and Nolen-Hoeksema, 2012; Aldao et al., 2010). The *adaptive approaches* are thought to promote wellbeing and are negatively associated with psychopathology (e.g., problem-solving and acceptance). In contrast, *maladaptive approaches* are believed to hinder wellbeing and are linked to psychopathology (e.g., avoidance and suppression). In the current work, we examined potentially adaptive versus maladaptive strategies in the context of climate change. Specifically, we were interested in two goals for managing affective responses to climate change: first, to manage personal wellbeing, and second, to promote climate action. We believe both are necessary to make climate change efforts sustainable in the long run.

Given that the realities of climate change can bring a variety of emotional reactions in the community, what do we know about effective ways of dealing with climate change? Very little is understood about effective ways people regulate their emotions, specifically in the context of climate change. A few researchers have discussed aspects of regulatory efforts that may arise in response to the climate change crisis. Building on the classic coping framework (Carver et al., 1989; Lazarus and Folkman, 1984), Reser and Swim (2011) proposed a two-phase model for understanding psychological coping with climate change. They argued that, as a first phase, threat appraisal involves assessing the impacts on oneself, family, community, culture, and society, alongside available resources, to determine risk probability and vulnerability. The second phase, coping appraisal, evaluates possible responses and the capacity to respond, considering self-efficacy, response efficacy, cost–benefit analysis, constraints, and the strength of external support. Others have also utilized the Lazarus & Folkman model to categorize regulatory approaches into three categories: problem-focused, emotion-focused, and meaning-focused (Homburg et al., 2007; Jovarauskaite and Böhm, 2020; Ojala, 2012b; Ojala, 2013; Zaremba et al., 2022; Carver, 2020). Accordingly, some people engage in *problem-focused* coping, which is generally considered adaptive and involves seeking information, social support, and undertaking personal mitigation and adaptation actions. In contrast, people can engage in *emotion-focused* coping, which is used to alleviate negative emotions through distancing oneself from the issue, denial, de-emphasizing the risks, or adopting an apathetic stance toward climate change. Emotion-focused coping may lead to apathy and denial, hindering willingness to engage in pro-climate action (Davidson and Kecinski, 2022; O'Neill and Nicholson-Cole, 2009; Smith and Leiserowitz, 2014; Zaremba et al., 2022). Another process, *meaning-focused* coping, involves regulating negative emotions through reframing perspectives, finding meaning, revising personal habits, or turning to spiritual comforts (e.g., Bradley et al., 2014). Meaning-focused coping is considered a generally adaptive approach to managing emotions, which may not necessarily extend to the context of climate change.

Specifically, in the climate change context, qualitative interviews (e.g., Zaremba et al., 2022) have found that people adopt a wide variety of regulation efforts to manage their emotional experiences. A recent cluster analysis found that individuals who were solution-focused addressed the climate crisis through a combination of problem- and meaning-focused strategies, whereas individuals who were avoidant utilized meaning-focused strategies and de-emphasized the climate risks (Rikner Martinsson and Ojala, 2024). Thus, individuals are shown to adopt a combination of strategies to deal with climate change. Following this literature, a recent framework (Kovacs et al., 2024) has also suggested the role that affect regulation strategies play in motivating climate action. They identified several affect regulation strategies that moderate climate action, such as shifting focus, rumination, other blame, and acceptance (Kovacs et al., 2024). Furthermore, other frameworks, such as Stern’s coherent theory of environmentally significant behavior (Stern, 2000), posit that engaging in pro-environmental behaviors is a complex interaction between causal variables (e.g., environmental dispositions, personal norms, perceived costs, and benefits), personal capabilities (e.g., literacy, resources, specific knowledge, and skills), contextual factors (e.g., laws, available technology, social norms, supportive infrastructure), and current routines. Similarly, Crandon et al. (2024) have argued that experiences of climate anxiety are not necessarily maladaptive and have recommended that other measures of regulatory efforts targeting climate change emotions are needed to better understand adaptive and maladaptive approaches. Together, the above frameworks suggest that climate change-related regulatory processes do not occur in isolation; instead, they are multifaceted and are informed by a combination of cognitions, personal beliefs, attitudes, behaviors, and socio-environmental factors.

### 1.2 Integrating affect regulation approaches to manage reactions to the climate crisis

In our integration of the literature on affect regulation and climate change, we identified several approaches that can be broadly discussed in three categories and are theoretically informed by existing frameworks: *proactive* (e.g., Carver et al., 1989; Lazarus and Folkman, 1984; Ojala, 2013; Reser and Swim, 2011), *reactive* (e.g., Carver et al., 1989; Lazarus and Folkman, 1984; Zaremba et al., 2022; Reser and Swim, 2011), and *stress-relief strategies* (e.g., Carver et al., 1989; Lohani et al., 2025b; Lohani et al., 2025a; Lohani et al., n.d.-b; Lohani et al., n.d.-a). We have highlighted instances where existing theoretical literature directly informs an affect regulation approach considered relevant in the context of climate change. In the following section, we organize the main strategies of interest that are pertinent to understanding regulatory efforts around climate change.

#### 1.2.1 Proactive regulation

*Proactive regulation* broadly encapsulates active engagement in managing emotions, especially by engaging with the climate change challenges (Reser and Swim, 2011). Proactive strategies are also similar to Lazarus and Folkman’s problem-focused and meaning-focused strategies, as well as planning and active coping, as outlined in Carver et al.’s framework. In the context of climate change, eco-consciousness has been frequently identified as a proactive approach. *Eco-consciousness* includes recognizing the causes and consequences of climate change and actively trying to do something about it (Lohani et al., 2025a; Lohani et al., 2025b). At its core, eco-consciousness is a collection of views that maintains the rights of all living things, including nature, to be respected and protected (Dunlap et al., 2000; Sanchez and Lafuente, 2010). Of particular significance to eco-consciousness are a person’s environmental worldviews and the importance of humans, nature, and the possibility of an eco-crisis (De Groot and Steg, 2007; Dunlap et al., 2000; Kollmuss and Agyeman, 2002). This includes strategies to gain objective knowledge about the dangers of climate change (Reser et al., 2012; Zaremba et al., 2022) and the causes of global warming (Bord et al., 2000; O’Connor et al., 2002). It also involves connecting with one’s environment (Reser et al., 2012). These cognitive processes go hand in hand with having a common concern for all (Zaremba et al., 2022) and identifying who contributes to the climate change crisis, including oneself and others (Kovacs et al., 2024; Zaremba et al., 2022). This can be associated with feeling alarmed about the climate crisis (Chryst et al., 2018) as well as frequent reflection on the state of the climate change crisis (Duchi et al., 2020; Verlie, 2019). Such concerns may also generate productive thought processes, such as feeling moral engagement, trying to problem-solve, and “doing your part” (Park and Ha, 2012).

Not surprisingly, individuals with significant eco-consciousness are more likely to participate actively in pro-climate action (Gkargkavouzi et al., 2018; Mathur and Kumari, 2013; Nisbet, 2009). Furthermore, environmentally conscious people are mindful of the effects of human actions on the environment. They strive to minimize actions that have negative impacts (Mathur and Kumari, 2013). Drawing from the affect regulation literature, problem-solving cognitive processes may include planning ahead or taking actions to address these challenges (Carver et al., 1989; Lohani et al., 2023). For example, individuals may feel motivated to solve problems (Park and Ha, 2012) and engage in sustainable behaviors (Bronfman et al., 2015; Reser et al., 2012). An example of targeting social networks is talking to your social circle to promote sustainability (Jones and Lucas, 2023). Problem-solving efforts can foster a positive outlook for the future (Dietz et al., 2007) and enhance environmental efficacy (Li and Monroe, 2019; Ojala, 2013), helping individuals—including young people who represent future generations—feel more prepared and empowered (Kelly et al., 2022; McMichael and Lindgren, 2011; Zaremba et al., 2022). Related strategies to problem-solving entail engaging in various active coping approaches, such as organized activism, eco-advocacy, and volunteerism, to mitigate climate issues (Herman, 2018; Zaremba et al., 2022). Therefore, several potential affect regulation approaches exist for individuals to effectively regulate affect and sustainability efforts in the face of climate challenges.

Another proactive approach is hope, a way of discovering solutions to tackle the challenges of climate change. *Hope* entails believing in the possibility of positive future climate outcomes achievable through collective actions, including individual efforts and more powerful societal initiatives (Janney et al., 2025; Ojala, 2012a, 2015; Lohani et al., 2025b; Lohani et al., 2025a; Lohani et al., n.d.-a). Although classic affect regulation and coping models do not directly refer to hope, climate scientists have discussed hope as a means to manage climate distress (e.g., Ojala, 2012a, 2015). It is akin to a growth mindset, reflecting optimism about the potential to change things about the world (Duchi et al., 2020). However, hope also acknowledges the inevitability of consequences and the uncertainty surrounding climate change resolution (Janney et al., 2025; Ojala, 2015; Ojala, 2012b; Ojala, 2012a; Ojala, 2023; Ojala, 2016; Zaremba et al., 2022; Zummo et al., 2025). Encouraging constructive hope proves pragmatic and advantageous in combating climate change. For most people, hope can entail the development and implementation of active pathways toward addressing climate change, in line with personal attributes to promote sustainability efforts (Ojala, 2015, 2023; Snyder, 2000).

#### 1.2.2 Reactive regulation

Unlike proactive forms of regulation, reactive regulation entails unhelpful ways of managing one’s responses to climate change, which can vary widely across individuals depending on their personal perceptions of the climate change crisis (based on Reser and Swim, 2011 model of climate change). Some approach the topic with apathy, while others may experience it as an unmanageable crisis. Below, we describe several types of approaches that may sometimes help people feel better, but they may be unhelpful in facilitating climate action. *Apathy* involves cognitions and behaviors of disbelief and indifference toward science-based facts about climate change (Lohani et al., 2025b). It includes cynicism and skepticism toward the scientific consensus on anthropogenic contributions to climate change (Ding et al., 2011). Related to apathy are any efforts to belittle, minimize, or trivialize the trajectory of climate change (Ding et al., 2011). Another aspect of apathy is being detached, unconcerned, or neglectful of the urgency and extent of climate change (Davidson and Kecinski, 2021). A distinct form is emotional denial, which has been recognized in the classic coping framework (Carver et al., 1989) and entails difficulty in processing or managing any emotions related to climate change (Reser and Swim, 2011).

In contrast to apathy, people may also experience dysregulated emotions due to climate change, as suggested by previous models, including reactive regulation (Reser and Swim, 2011) and the emotion-focused coping model (Lazarus and Folkman, 1984). One of the extreme approaches can lead to feelings of *doom,* which includes perceptions that climate change problems are too big and unavoidable, rendering one’s efforts to make a difference as meaningless. A lack of effective ways to deal with the climate change crisis may make it feel like a lost cause (Zaremba et al., 2022). The inevitability of climate change can cause feelings of helplessness and powerlessness in the ability to do something, resulting in climate despair and a lack of action (Reser et al., 2012).

Another form of dysregulation is *conflict,* which includes struggles to align one’s behaviors with one’s beliefs on climate change (i.e., cognitive dissonance). *Although* there is no classic model that directly captures it, recent work has acknowledged that people may experience conflict due to climate change challenges (e.g., Ágoston et al., 2022; Clayton and Karazsia, 2020). For example, failing to act in a climate-friendly manner despite believing in climate change can generate feelings of inadequacy, self-blame, and despair (Ágoston et al., 2022). Related strategies are wishful thinking (Clayton and Karazsia, 2020) and procrastination regarding climate-friendly activities for sustainability.

Another dysregulated emotional effort entails dealing with feeling like an *outcast,* which can arise when individuals feel socially isolated and alienated in their personal perspectives on climate change, leading to a need to separate from others (Zaremba et al., 2022). As in classic affect regulation and coping literature, seeking social support is an important way of managing one’s distress (Carver et al., 1989; Folkman et al., 1986). While social support can provide a strong buffer, a lack of social support can generate feelings of impairment and an inability to engage in open conversations (i.e., *stonewalling*; Choo, 2017; Hornsey and Fielding, 2019) or reliance on substance abuse activities (Carver et al., 1989; Tomassini et al., 2024) to cope with intense dysregulated emotions around climate change.

A common unhelpful strategy in affect regulation and coping literature is *withdrawal* (and forms of avoidance), when individuals can adopt cognitive and behavioral approaches to avoid dealing with climate change (Lohani et al., 2025a). It is similar to mental and behavioral disengagement in Carver’s coping model and escape-avoidance in Lazarus and Folkman’s framework. Accordingly, in the context of climate change, an example is information avoidance (Zaremba et al., 2022), which involves efforts to disengage from climate change-related details. Another strategy is *experiential avoidance* (Bradley et al., 2014; Kovacs et al., 2024; Lohani et al., 2025b; Lohani et al., 2025a), which entails ignoring emotions related to climate change. Efforts can also be made to explicitly *suppress* any experience or display of emotions in response to climate change thoughts or conversations (Bloodhart et al., 2019; Kovacs et al., 2024; Norgaard, 2006). Another related approach can be a failure to accept (Lohani et al., 2020) the emotional significance of climate change. As such, individuals could also do something else to focus away from the climate change crisis and on themselves. *Self-distraction* (Armstrong et al., 2025; Kovacs et al., 2024; Lohani et al., 2022; Lohani et al., 2025a; Lohani et al., n.d.-b; Lohani et al., n.d.-a) away from engaging with climate change challenges and instead focusing on something else, such as exercising or spending quality time with others, could help reduce negativity. At other times, individuals may withdraw because they lack the resources or skills necessary to actively address the climate crisis, leaving them with limited coping options (Davidson and Kecinski, 2021). These efforts are generally targeted at managing one’s emotions around climate change, with varied success in supporting one’s wellbeing.

#### 1.2.3 Stress-relief approaches

In addition to proactive and reactive forms, the literature on classic affect regulation strategies suggests several stress-relief approaches people may adopt to regulate their affect. *Spiritual-bodily practices* can include a variety of behaviors and cognitive approaches to achieve this goal. They encapsulate the idea of finding comfort and calm through spiritual practices—such as religion, prayer, and meditation—to seek support and recover from negativity (Lohani et al., 2025b). For instance, classic literature on coping has talked about finding comfort in religion (Carver et al., 1989) or reducing stress by immersing oneself in nature (Berto, 2014). Such stress-relief practices can also promote climate action (Pihkala, 2022b). Spirituality has also been considered a meaning-focused coping (Ojala, 2012b). Indeed, a recent study found that community members (Lohani et al., 2025b) and students frequently utilized religious practices to regulate their affect and manage climate distress (Lohani et al., 2025a). Furthermore, religious responses to climate change that frame humans as part of nature have been suggested to promote hope and engagement (White, 2018). Another non-spiritual approach involves exercise and relaxation techniques, which have been shown to be effective in regulating climate distress (Bernard et al., 2022). Indeed, a recent study revealed that students learning about climate change found exercise, yoga, and other relaxation techniques to be effective in dealing with climate change stressors (Lohani et al., 2025a).

In contrast, the use of humor as a stress-relief strategy can help neutralize negative emotions. *Humor* (or overplaying) can help make an extremely difficult situation more manageable, including climate change (Carroll-Monteil, 2023; Christie and Moore, 2005; Kaltenbacher and Drews, 2020). Humor can be an avoidant regulation strategy used to minimize a problem for momentary emotional relief (Simione and Gnagnarella, 2023). In the context of climate change, humor has been found to decrease the perceived risk of the climate crisis (Skurka et al., 2018), raising concerns about its potential to disrupt pro-climate action. However, humor can also be a more active or approach-oriented coping strategy that prioritizes long-term wellness by reframing a stressor to make it more manageable (Simione and Gnagnarella, 2023). This is similar to cognitive reappraisal (also considered a kind of meaning-focused coping; Ojala, 2012b), which focuses on changing the appraisal of the situation to deal with the climate crisis (Kovacs et al., 2024). Another frequently utilized affect regulation strategy involves modifying potentially stressful situations (Sheppes et al., 2015). In the context of climate change, relocating could be a behavioral approach to managing climate challenges (Seebauer and Winkler, 2020; Stone, 2012; Thornton et al., 2014). Overall, all the self-soothing approaches are focused on improving or maintaining wellbeing, with a feeling of hope, particularly supporting climate action (Janney et al., 2025; Lohani et al., 2025b; Lohani et al., n.d.-a; Zummo et al., 2025).

### 1.3 The current study

Following the literature reviewed thus far, the current work aimed to gain a thorough understanding of the possible regulatory approaches targeting climate change. Given the diverse nature of strategies employed, it was necessary to examine a broad range of both cognitive and behavioral approaches to regulate affective responses toward climate change. However, one existing framework did not capture this content, and thus integration from several models was necessary. In the current research, we expand on existing frameworks that we found relevant to this conversation, adopting a more exhaustive approach by drawing on traditional affect regulation *and* climate change literature. These strategies extend beyond those commonly identified in previous literature discussed thus far. The overarching goal of the current research was to develop a comprehensive understanding of how people manage their responses to the pressing issue of climate change. To achieve this goal, we developed an inventory to measure common approaches people may use to manage their reactions to climate change.

Based on the background literature discussed in *Sections* 1.1 and 1.2 (e.g., Homburg et al., 2007; Jovarauskaite and Böhm, 2020; Lazarus and Folkman, 1984; Ojala, 2013; Reser and Swim, 2011; Zaremba et al., 2022), several strategies were identified with the expectation that they could overlap. Given the research gap with no single existing framework capturing diverse affect regulation strategies in the context of climate change, we adopted a multiple-phase approach that included starting from a much larger number of strategies and then, based on factor analysis metrics, retaining the more relevant strategies. To do this, best practices from exploratory factor analysis were adopted after data collection to determine the underlying structure of affect regulation in the context of climate change (Costello and Osborne, 2005; Kline, 2015; Schreiber et al., 2006; Tabachnick and Fidell, 2019). This process of scale development led to the *ECO-SHADOW inventory*, an acronym that captures the nine factors that were found—Eco-consciousness, Conflict, Outcast, Spirituality, Hope, Apathy, Doom, Overplay, and Withdrawal. Following the protocol for new inventory development, we also conducted reliability and validation steps; details are provided in the *Results section*.

## 2 Methods

### 2.1 Participants

A total of 594 participants with a mean age of 21.9 years (age range was 18–48 years old) completed this study. Of these, 67.68% were females, 24.92% were male, 1.3% were transgender, and 2.4% reported other. The ethnic breakdown was as follows: 0.84% American Indian/Alaskan, 1.18% African American, 8.75% Asian, 66.67% Caucasian, 8.25% Multiracial, 0.51% Native Hawaiian, 3.20% Pacific Islander, and 9.09% others. Convenience sampling was used to collect data from the university’s participant pool, resulting in a relatively decent sample of young adults (the population of interest) enrolled in an undergraduate degree at the University of Utah.

Two attentional checks were included within the surveys to ensure that participants were engaged in the survey. If participants got either wrong, we removed their data completely and did not utilize it for the analysis reported in the *Results* section. Initially, we had 594 participants, and after removing 4.71% of participants who failed the attentional check, 566 participants remained. No specific exclusion criteria were applied beyond attention check failure. Our sample size was guided by common recommendations for factor analysis (e.g., 10 participants per item), ensuring adequate power for the planned exploratory factor analysis.

### 2.2 Measures

Existing measures were included to examine the validity of the developed ECO-SHADOW inventory. The following established measures were used to evaluate convergent and divergent validity for the independent factors within the inventory.

#### 2.2.1 Climate distress and interference scale

The Climate Distress and Interference Scale (C-DIS; Guan et al., 2024) adapts the Acceptance and Action Questionnaire (AAQ-II; Bond et al., 2011) to measure climate distress and experiential avoidance and psychological inflexibility in the context of climate change (Guan et al., 2024). The C-DIS shortens the AAQ-II to 7 items, which focus on managing climate-related distress. The questionnaire has a maximum summed score of 24 and a minimum of 0, with higher scores indicating greater climate-related distress and experiential avoidance (Guan et al., 2024). In the current study, the C-DIS had a mean of 1.9 (*SD* = 0.85) and good reliability, and a Cronbach’s alpha of 0.89.

#### 2.2.2 Screener for climate anxiety and depression

The Screener for Climate Anxiety and Depression (Uppalapati et al., 2023) was used to assess anxiety and depression symptoms related to global warming. It modifies the two items of the Generalized Anxiety Disorder subscale (GAD-2; Kroenke et al., 2007) and the Patient Health Questionnaire (PHQ-2; Kroenke et al., 2003) depression screener. The sum of the first two items can range from 0 to 6, with a 3 or higher indicative of a generalized anxiety disorder related to global warming. The sum of the last two items can range from 0 to 6, with a 3 or higher indicative of depression related to global warming. In the current study, this measure had a mean of 1.2 (*SD* = 0.41), good reliability, and a Cronbach’s alpha of 0.82.

#### 2.2.3 New ecological paradigm

The Revised New Ecological Paradigm (NEP; Dunlap et al., 2000), improved from Dunlap & Van Liere’s New Ecological Paradigm (Dunlap and Van Liere, 1978), is an instrument that measures environmental concern and beliefs about humanity’s relationship with nature using 15 Likert items (De Groot and Steg, 2007). The New Ecological Paradigm is a perspective that emphasizes the interdependence of humanity and nature. It represents an ecological worldview that asserts that humans should coexist harmoniously with nature and have a responsibility to maintain a balance between the environment and anthropogenic change. This perspective is indicated through agreement with the odd-numbered items on the NEP instrument. The NEP scale had a mean of 4 (*SD* = 0.6) and a Cronbach’s alpha of 0.72.

By contrast, the Dominant Social Paradigm (DSP) suggests that humans are dominant over nature and, therefore, have the right to use and control nature for societal advancement. From this perspective, environmental damage is justified if it leads to technological breakthroughs or economic growth, which is indicated by agreement with the even-numbered items on the NEP instrument (Anderson, 2012). The DSP scale had a mean of 2.7 (*SD* = 0.71) and a Cronbach’s alpha of 0.74.

#### 2.2.4 Six Americas short survey

The Six Americas Short Survey (SASSY; Chryst et al., 2018) divides Americans into six distinct audience segments based on beliefs, attitudes, and behaviors regarding climate change. The six groups include Alarmed, Concerned, Cautious, Disengaged, Doubtful, and Dismissive and are placed on a spectrum where the Alarmed group represents Americans who have the highest belief in and concern about climate change, as well as the strongest motivation to engage in mitigative behaviors. On the opposite end, the Dismissive group consists of Americans with the lowest belief in and concern for climate change paired with active opposition to mitigative behaviors. The original 36-item survey (Maibach et al., 2011), previously condensed into the 15-item reduced discriminant model tool, was further reduced to just four items (Chryst et al., 2018) by identifying and verifying the top four variables. SASSY was demonstrated to successfully divide Americans into six groups with an accuracy rate of about 70% (Chryst et al., 2018). SASSY had a mean of 2.9 (*SD* = 1.3) and good reliability, with a Cronbach’s alpha of 0.81.

#### 2.2.5 Barratt impulsiveness scale–brief

The Barratt Impulsiveness Scale (Barratt, 1975; Coutlee et al., 2014) measures attentional, motor, and non-planning impulsiveness. Through the use of factor analysis, the original 30-item scale was reduced to 13 items that were most effective at measuring the three types of impulsiveness (Coutlee et al., 2014). The items are measured on a four-point Likert scale with a composite score range of 13 to 52. After reverse coding relevant items, a higher score represents greater impulsivity, while a lower score represents lower impulsivity. Three sub-scales can be calculated—attention, motor, and non-planning. *The attention* subscale (*M* = 3.1, *SD* = 0.57, Cronbach’s alpha = 0.73) assesses the inability to pay attention, measured using five items. Sub-scores range from 5 to 20, with higher scores after reverse coding indicating greater attention impulsivity. *Motor* subscale (*M* = 2, *SD* = 6.8, Cronbach’s alpha = 0.84) measures acting without thinking, using four items. Sub-scores range from 4 to 16, with higher scores after reverse coding indicating greater motor impulsivity. *Non-planning* subscale (*M* = 3.2, *SD* = 0.7, Cronbach’s alpha = 0.82) assesses the inability to plan ahead for the future, measured using four items. Sub-scores range from 4 to 16, with higher scores after reverse coding indicating greater non-planning impulsivity.

#### 2.2.6 Positive and negative affect scale

A modified version of the Differential Emotion Scale (Watson and Clark, 1994; Watson et al., 1988) was used to measure the experience of positive and negative affect in response to climate change (Lohani et al., 2025b; Lohani et al., n.d.-b). Participants were informed: “Thinking about climate change may or may not be emotionally challenging. Does thinking about climate change make you feel any of the following?” The negative words included anxious, grief, angry/outraged, disgusted, depressed, sad, hurt, afraid/scared, hopeless, ashamed, frustrated, powerless, guilty, worried, stressed, and despair. The positive words included optimistic, inspired, happy, hopeful, and interested. Participants rated each emotional word on a scale of *Not at all* to *Extremely*. An average score for each category of words was calculated. The negative affect had a mean of 2.7 (*SD* = 1.1) and a Cronbach’s alpha of 0.96, while the positive affect had a mean of 2.00 (*SD* = 0.73) and a Cronbach’s alpha of 0.75. The averaged score for negative and positive words assesses unpleasant and pleasant feelings related to climate change, respectively.

### 2.3 Item selection for the ECO-SHADOW measure

To establish the content validity of the initial item pool, we employed a multi-step, qualitative process grounded in theory and expert judgment. Item generation began with an extensive review of the literature. Several approaches were adopted to identify items to assess different strategies relevant to how people may process and cope with climate change. These strategies included beliefs, values, cognitions, behaviors, and socio-environmental factors. To create a comprehensive measure, first, items were drawn from existing measures, including some existing items (Ahmed et al., 2015; Carver et al., 1989; Folkman et al., 1986; Garnefski and Kraaij, 2006; Grommisch et al., 2020; Kalokerinos et al., 2017; Medland et al., 2020; Nisbet and Zelenski, 2013; Ojala, 2012b; Ojala, 2012a; Radakovic et al., 2020; Raes et al., 2011; Reser et al., 2012; Thompson and Barton, 1994; Valquaresma et al., 2022). Second, other items were drawn from neutral contexts and adapted to the climate change context. That is, if items for a more generic context existed (e.g., “*When I feel inadequate in some way, I try to remind myself that feelings of inadequacy are shared by most people*” from Self-Compassion Scale – Short Form; Raes et al., 2011), they were adapted to the climate change context, (e.g., “*When I feel inadequate in dealing with climate change challenges, I try to remind myself that feelings of inadequacy are shared by most people*”). Similarly, when the items were from another specific context (e.g., “*S/he is indifferent to what is going on around him/he*”; see the Brief Dimensional Apathy Scale; Radakovic et al., 2020), they were adapted to the climate change context (e.g., “*I’ve been feeling indifferent to what is going on with climate change*”). Third, when no existing items were available for several strategies, those items were created. This process informed the development of an initial pool of items intended to capture the core dimensions and nuanced aspects of the construct. Items were written to be conceptually clear, theoretically grounded, and appropriate for the target population.

Following item generation, three subject matter experts—including researchers in affect regulation and climate change—reviewed the item pool. These experts assessed each item for conceptual relevance, clarity, representativeness, and potential redundancy. Rather than relying on quantitative indices, we used a qualitative approach to emphasize rich, contextualized feedback and theoretical alignment. Expert feedback was collected via written commentary and collaborative discussions. Pilot testing was conducted with five individuals to get qualitative feedback on items in terms of fit and clarity. Based on this input, items were revised, removed, or refined across two rounds of review. Accordingly, items were revised and improved before starting data collection for psychometric purposes, which is reported in the current manuscript. The current work focused on ensuring comprehensive coverage of constructs and eliminating redundancy. Additionally, we refined language clarity and ensured that items were understandable and appropriate for the intended respondent group. This iterative process enhanced the conceptual coherence and content validity of the final item set used in the subsequent empirical phases of the study.

The total number of original items was 124 (as some strategies had more than one item associated with them). Fourth, we utilized psychometric information to refine the items that capture the construct of affect regulation in the climate change context. This included the removal of items that were below recommended thresholds and the retention of items that contributed to the inventory. Additional item processing and data analysis steps are outlined in *Sections 2.5 and 3.2*, which describe the statistical approaches used to reduce the final number of items to 65; Table 1 presents the individual items of the ECO-SHADOW inventory. Following the removal of less relevant items, the remaining items provided a more reliable representation of the underlying construct.

**Table 1:** The ECO-SHADOW items with factor loadings (FL), communalities (*h*^2^), and unique variance (*u*^2^).

#	Strategy descriptor	Item	FL	*h* ^2^	*u* ^2^
Factor 1: Eco-consciousness					
1.01	Information seeking	I’ve been searching for information about what I as an individual can do.	0.84	0.68	0.32
1.02	Problem-focused-coping self	I’ve been thinking about what I myself can do.	0.82	0.68	0.32
1.03	Problem-focused-coping social	I’ve been talking with my family and friends about what one can do to help.	0.78	0.59	0.41
1.04	Planning	I’ve been thinking hard about what steps to take.	0.75	0.62	0.38
1.05	Eco-advocacy	I’ve been encouraging people around me to become more aware of environmental issues.	0.71	0.59	0.41
1.06	Active-coping	When considering the challenges of climate change, I feel it is important to look for things that I can address and change in my everyday life.	0.67	0.62	0.38
1.07	Rumination	I’ve been thinking about the climate change issues again and again.	0.54	0.62	0.38
1.08	Eco-consciousness cognitive	Climate change has forced me to change the way I think about and view how we live in and use our natural environment in [state]/region.	0.52	0.6	0.4
1.09	Sustainable-behavior	I’ve been trying to reduce my behaviors that contribute to climate change.	0.52	0.39	0.61
1.1	Reflection	I’ve been continually thinking about what was bothering me, specifically about climate change.	0.48	0.6	0.4
1.11	Connect with Environment	I always think about how my actions affect the environment.	0.46	0.4	0.6
1.12	Significance	I’ve been thinking about all the different things in my life that climate change would impact.	0.45	0.54	0.46
1.13	Moral-engagement	I feel a moral duty to do something about climate change.	0.42	0.5	0.5
1.14	Volunteerism	I’ve been trying to connect with efforts that would help address climate change.	0.41	0.46	0.54
1.15	Environmental-efficacy	I’ve been feeling that I can do something about climate change.	0.4	0.39	0.61
1.16	Cognitive-reappraisal	I’ve been thinking of other ways to interpret the climate change problems.	0.38	0.34	0.66
1.17	Concerned	It has been upsetting me that there seems to be so little that I can do to address environmental problems such as climate change.	0.36	0.57	0.43
1.18	Change-of-habitat	I have seriously thought about alternative places to live because of the increasingly evident impacts of climate change.	0.36	0.33	0.67
Factor 2: Conflict					
2.01	Cognitive dissonance (behavioral)	When it comes to climate change, I feel that my thoughts and actions are contradictory.	0.52	0.39	0.61
2.02	Cognitive dissonance (cognitive)	I’ve been experiencing mental conflict because my climate change beliefs do not line up with my actions.	0.51	0.39	0.61
2.03	Ambivalence	Even though I believe in climate change, I do not feel that I am able to act sustainably and live an environmentally friendly life.	0.48	0.41	0.59
2.04	Wishful-thinking	I’ve been wishing I behaved more sustainably.	0.46	0.49	0.51
2.05	Procrastination	I’ve been putting off doing environmentally friendly behaviors that can be done.	0.36	0.26	0.74
2.06	Self-blame	I’ve been blaming myself for issues happening with climate change.	0.33	0.38	0.62
Factor 3: Outcast					
3.01	Isolated	I’ve been feeling alone and isolated in my concerns about climate change.	0.62	0.5	0.5
3.02	Social exclusion	I’ve been feeling left out because of my beliefs about climate change.	0.62	0.43	0.57
3.03	Impairment	My concerns about climate change have been undermining my ability to get work done up to my potential.	0.45	0.43	0.57
3.04	Substance-abuse	I’ve been using alcohol or other drugs to make myself feel better about climate change problems.	0.43	0.27	0.73
3.05	Alienated	I’ve been engaging in environmental volunteerism.	0.43	0.37	0.63
3.06	Stonewalling	I’ve been refusing to have a discussion about climate change.	0.43	0.3	0.7
3.07	Hesitation	I’ve been hesitant to share my true feelings about climate change with others.	0.42	0.32	0.68
Factor 4: Spiritual-bodily practices					
4.01	Religious belief	I’ve been trying to find comfort in my religion or spiritual beliefs.	0.92	0.84	0.16
4.02	Prayer-meditation	I’ve been praying or meditating.	0.87	0.77	0.23
4.03	Relaxation	I’ve been trying to take deep breaths or exercise.	0.34	0.3	0.7
Factor 5: Hope					
5.01	Hope–Scientists	I have faith in scientists and people engaged in environmental organizations to come up with a solution in the future.	0.72	0.53	0.47
5.02	Hope–Humanity	I have faith in humanity; I believe we together can do something about climate change.	0.71	0.56	0.44
5.03	Hope–Solution	I’ve been thinking that the climate change problem will be solved in the future.	0.71	0.51	0.49
5.04	Rational Optimism	I’ve been thinking that even though climate change is a big problem, one has to have hope.	0.65	0.5	0.5
Factor 6: Apathy					
6.01	Downplay	I’ve been thinking that the climate change threats have been exaggerated.	0.78	0.65	0.35
6.02	Apathy	I do not care about climate change.	0.67	0.48	0.52
6.03	Belittle	I’ve been thinking that the problem of depletion of natural resources is not as bad as many people make it out to be.	0.67	0.41	0.59
6.04	Irrelevance	I cannot be bothered to care about climate change.	0.65	0.47	0.53
6.05	De-emphasizing	I feel that nothing serious will happen during my lifetime.	0.65	0.47	0.53
6.06	Perspective-taking	I’ve been thinking that the climate change problem has not been too bad compared to other things.	0.57	0.46	0.54
6.07	Indifference	I’ve been feeling indifferent to what is going on with climate change.	0.56	0.38	0.62
6.08	Skepticism	I’ve been thinking that climate change is due to Earth’s natural cycles, so humans can have little influence on it.	0.48	0.34	0.66
6.09	Low-priority	I’ve been thinking that our society has many other challenges that need to be prioritized.	0.43	0.33	0.67
6.10	Denial (emotional)	I say to myself, “This is not real.”	0.41	0.31	0.69
Factor 7: Doom					
7.01	Overwhelmed	I’ve been thinking that climate change problems seem too big for me to do anything about.	0.86	0.75	0.25
7.02	Purposeless	I’ve been thinking that my efforts on climate change are too small to make a difference.	0.82	0.68	0.32
7.03	Meaningless	I feel there is little meaning in the things I do, and it will not make a difference in addressing climate change.	0.53	0.4	0.6
7.04	Limited	I’ve been thinking that it is not that I do not care, I just have a lot of other challenges to deal with.	0.43	0.32	0.68
7.05	Powerless	I’ve been feeling helpless and I do not know how to overcome the climate change problem.	0.41	0.53	0.47
7.06	Lost-cause	I think the environmental destruction is unavoidable and I do not think that things will get better.	0.38	0.34	0.66
7.07	Other-blame	I’ve been feeling that the cause of climate change lies with others, such as policymakers, corporations, people in power, and the media.	0.35	0.38	0.62
Factor 8: Overplay					
8.01	Humor	I’ve been making fun of the situation.	0.93	0.87	0.13
8.02	Mocking	I’ve been making jokes about it.	0.88	0.79	0.21
Factor 9: Withdraw					
9.01	Experiential avoidance	I’ve been trying to avoid thinking about climate change-related feelings and problems.	0.54	0.46	0.54
9.02	Worry (universal)	I’ve been worrying about myself, my relatives, future generations, animals, and nature	0.47	0.63	0.37
9.03	Self-distraction	I’ve been turning to work or other activities to take my mind off issues related to climate change.	0.44	0.36	0.64
9.04	Catastrophizing	I’ve been continually thinking how horrible the situation is.	0.44	0.37	0.63
9.05	Information avoidance	I’ve been trying to avoid information about climate change issues.	0.37	0.54	0.46
9.06	Nonacceptance	I’ve been unable to accept how bad the climate change problems have become.	0.37	0.28	0.72
9.07	Suppression	I’ve been making an effort to hide my feelings.	0.33	0.38	0.62
9.08	Behavioral Self-distraction	I’ve been doing something to think about climate change less, such as going to movies, watching TV, reading, daydreaming, sleeping, or shopping.	0.33	0.2	0.8

### 2.4 Procedure

All procedures for this study were in accordance with the Institutional Review Board of the University of Utah. Before starting the study, all participants provided their written informed consent by signing the approved consent form. Based on existing work, items for the new measure were generated or adapted for the context of climate change.

Before participants started filling out the ECO-SHADOW inventory, respondents first read an introductory text that described some facts about climate change specific to the state where the responders lived (see Appendix). The purpose of this introductory text was to ensure all participants were thinking about climate change before they responded to the ECO-SHADOW inventory. Participants read the following introduction: *Thinking about the impacts of [State’s/Region’s name] changing climate may or may not be emotionally challenging. The following survey aims to explore how people typically respond to climate change in their lives. Given, there are lots of ways, we are interested in your responses to climate change. The following sections of this survey will present several possible ways. We want to know to what extent you have been doing what the item says,* i.e.*, how much or how frequently.”* After participants completed the inventory, they also completed some existing measures to examine validity, followed by some demographic questions. Participants completed all questions online via a Qualtrics survey in one sitting. It took participants between 30 and 45 min to complete the entire study. The items were randomized across participants to reduce the effect of potential fatigue.

### 2.5 Data analysis plan

Given that we did not expect a fixed number of factors, Exploratory Factor Analysis (EFA) was conducted, and best practices suggested for this technique were followed (Costello and Osborne, 2005; Kline, 2015; Schreiber et al., 2006; Tabachnick and Fidell, 2019). The *psych* R package (Revelle and Revelle, 2015) was used to conduct EFA with principal axis factoring (which is robust to violations of normality assumptions; Fabrigar et al., 1999) and *promax* rotation (because affect regulation factors were expected to be correlated; Ford et al., 2019). We determined the eigenvalues and a scree plot to examine the potential number of factors. Factor loadings were analyzed using the *factanal* function in the *psych* R package. The significance level of *alpha* < 0.05 was set as the criterion. Several steps were followed to finalize the items within each factor. The factor loadings (FL) were reviewed for items that had low loadings (<0.3) or high cross-loadings. Any items that had factor loadings below the predetermined threshold were dropped from the model.

Next, Structural Equation Modeling (SEM) using *lavaan* (Rosseel et al., 2017) and *semPlot* (Epskamp et al., 2022) packages was conducted to compare the Initial model to the Reduced model after items were dropped to improve reliability. For validity testing, a method robust to multivariate assumptions was utilized, called the maximum likelihood robust (MLR) estimator in the *lavaan* package (Rosseel et al., 2017). An estimated latent correlational analysis was conducted to examine convergent and divergent validity; see Table 2.

**Table 2:** Correlations presented between the ECO-SHADOW (Eco-consciousness, Conflict, Outcast, Spirituality, Hope, Apathy, Doom, Overplay, and Withdrawal) scales and Convergent Validity measures.

ECO-SHADOW scale	C-DIS	Negative affect	Positive affect	SASSY	C-A + D	NEP odd	NEP even	Attention	Motor	Non-planning
E	0.37***	0.59***	0.21***	−0.56***	0.18***	0.23***	−0.18***	−0.01	0.04	0.03
C	0.37***	0.43***	0.11**	−0.25***	0.14***	0.17***	0.03	0.04	0.09	−0.05
Ou	0.30***	0.23***	0.09*	−0.13**	0.19***	0.03	0.02	0.02	0.04	−0.01
S	0.01	−0.003	−0.01	0.07	0.04	−0.06	0.03	0.02	0.11	−0.06
H	−0.01	0.01	0.27***	−0.04	−0.02	0.01	0.02	−0.02	−0.01	0.03
A	−0.16***	−0.33***	0.01	0.49***	−0.03	−0.31***	0.35***	−0.01	0.02	<0.001
D	0.36***	0.44***	−0.04	−0.25***	0.10*	0.23***	−0.19***	0.03	0.04	−0.03
Ov	0.004	−0.14**	0.18***	0.14***	0.02	−0.13**	0.04	−0.03	−0.05	0.09*
W	0.57***	0.76***	0.12**	−0.63*	0.22***	0.29***	−0.22***	0.02	0.05	0.02

***p < 0.001 (two-tailed), **p < 0.01 (two-tailed), *p < 0.05 (two-tailed).

Model comparisons helped select the final model. The reliabilities based on Cronbach’s alpha were examined, and the alignment of each item with the underlying construct as represented by the factor was evaluated. We also ensured that removing an item still maintained a factor’s reliability and practical relevance. SEM was also used to conduct validity comparisons with existing measures.

## 3 Results

### 3.1 Preliminary analysis

Before conducting EFA, the factorability of the dataset was assessed using the Kaiser-Meyer-Olkin (KMO) measure and Bartlett’s test of sphericity (Bartlett, 1954). The overall KMO value was excellent (KMO = 0.91), indicating that the sample was adequate for factor analysis (Kaiser, 1974). Additionally, the majority of items had individual KMO values above 0.80, suggesting strong item-level sampling adequacy; only two items fell below the generally accepted threshold of 0.70, indicating that they may contribute less to shared variance among the items. Bartlett’s test of sphericity was statistically significant, *χ^2^*(630) = 8436.80, *p* < 0.001, rejecting the null hypothesis that the correlation matrix is an identity matrix. Together, these results support the suitability of the data for conducting EFA. Among assumptions, normality was not met; therefore, we used principal axis factoring, which is robust to violations of multivariate assumptions than the Maximum Likelihood method (Fabrigar et al., 1999; Costello and Osborne, 2005).

### 3.2 Exploratory factor analysis

EFA was conducted to examine the eigenvalues, the scree plot, and determine the factor structure. To guide item retention during EFA, we followed widely accepted methodological guidelines. Specifically, items were retained if they exhibited a primary factor loading of at least 0.40, and items with cross-loadings ≥ 0.30 on more than one factor were excluded to ensure discriminant validity and factor clarity (Costello and Osborne, 2005; Tabachnick and Fidell, 2019). These threshold levels were selected to strike a balance between statistical rigor and the interpretability of the factor structure. Decisions regarding item retention were informed by both empirical criteria and theoretical alignment with the construct being measured. Inspection of the correlation matrix revealed many coefficients above 0.30, supporting sufficient inter-item correlations.

After dropping items that did not meet the 0.30 loading threshold, we evaluated two models using SEM: an Initial model including all items, and a Reduced model containing only items that exceeded the threshold. The *Initial model*, based on the original item set, generated suboptimal fit statistics, *χ^2^*(2439) = 7515.20, *p* < 0.0001, Akaike Information Criterion (AIC) = 103,161, Bayesian Information Criterion (BIC) = 103,980, and Root Mean Square Error of Approximation (RMSEA) = 0.061, indicating potential misspecification (Brown, 2015; Hu and Bentler, 1999). Guided by both Cronbach’s alpha and factor loadings, items with weak performance, below the threshold, were removed, resulting in the reduced model. This *Reduced model* demonstrated improved fit: *χ^2^*(1979) = 6294.27, *p* < 0.0001, AIC = 93,643, BIC = 94,363, and RMSEA = 0.062. These results suggest that the Reduced model with the refined item set more consistently captured the intended latent constructs.

The model comparison revealed a significant improvement in model fit with the Reduced model based on the metric comparisons: (1) Chi-square difference, *χ^2^*_Difference_(460) = 1,221, *p* < 0.0001; This indicated that the Reduced Model was significantly better than the Initial model, as the lower chi-square value indicates a better fit. (2) AIC_Difference_ = 9,518, a lower AIC indicates a better trade-off between model fit and complexity, favoring the Reduced Model. (3) BIC_Difference_ = 9,617; Like AIC, a lower BIC suggests that the Reduced Model is better when considering both model fit and complexity. (4) RMSEA_Difference_ = 0.054; The RMSEA value for the Reduced Model was lower than the Initial model, indicating a better model fit. Thus, based on these model-fit comparisons, the Reduced model with fewer items was a better fit for explaining the data than the Initial Model, and was chosen as the final model. The comparison of these models was helpful in refining our measurement tool to enhance their validity and reliability (Kline, 2015; Schreiber et al., 2006).

The means and standard deviations (SDs) for each of the scales of ECO-SHADOW are reported in Table 3. On a 5-point Likert scale, a mean of 3 suggests a balanced distribution of responses. Extremely high or low means may indicate ceiling or floor effects, which we did not observe in these data. An SD around 1.0 on a 5-point Likert scale is generally acceptable, implying a reasonable spread of responses. A very low SD could suggest that most respondents provided similar answers, which may reduce the variability needed for a reliable scale. The factor loadings and items are reported in Table 1. We removed items with factor loadings or cross-loadings below 0.3. The retained items, representing more than 60 strategies, were found to align with and be informed by theoretical literature. See the column “Strategy Descriptor” in Table 1 for the strategies represented by each retained item. The underlying factor structure best represented by the final affect regulation strategies is described below, and the intercorrelations are shown in Table 4.

**Table 3:** The Cronbach’s alphas for each of the scales of the ECO-SHADOW inventory are presented with the Mean (Standard Deviation) from a sample of college students (*N* = 566).

ECO-SHADOW scale	Cronbach’s alpha	Mean (SD)	# of items
Eco-consciousness	0.93	2.20(0.73)	18
Conflict	0.72	2.00 (0.63)	6
Outcast	0.75	1.40 (0.51)	7
Spirituality-bodily practice	0.73	2.00 (2.00)	3
Hope	0.80	2.9 (0.91)	4
Apathy	0.86	1.7 (0.66)	10
Doom	0.80	2.70 (0.85)	7
Overplay	0.82	1.71 (1.00)	2
Withdrawal	0.76	2.2 (0.74)	8

**Table 4:** Intercorrelations between the eight scales of the ECO-SHADOW inventory (*N* = 566).

ECO-SHADOW scale	1	2	3	4	5	6	7	8
1 Eco-consciousness	1							
2 Conflict	0.16**	1						
3 Outcast	0.24**	0.22**	1					
4 Spirituality-bodily	0.06	0.02	0.05	1				
5 Hope	0.23**	0.07	0	0.2**	1			
6 Apathy	−0.38**	−0.07	0.08	0.24**	−0.02	1		
7 Doom	0.06	0.33**	0.16**	−0.12*	−0.05	−0.07	1	
8 Overplay	0.05	0.14**	0.11**	0.04	−0.03	0.25**	0.19**	1
9 Withdrawal	0.36**	0.32**	0.28**	−0.06	0.06	−0.19**	0.28**	0.1*

***p < 0.001 (two-tailed), **p < 0.01 (two-tailed), *p < 0.05 (two-tailed). Sem for latent variables were used.

### 3.3 The finalized ECO-SHADOW factors

Based on the underlying strategies, the following nine ECO-SHADOW factors were identified (see Tables 1, 5 for details): (1) *Eco-consciousness* which is a proactive, problem-focused, and meaning-focused approach informed by past work (e.g., Bronfman et al., 2015; Carver et al., 1989; De Groot and Steg, 2007; Dietz et al., 2007; Duchi et al., 2020; Verlie, 2019; Dunlap et al., 2000; Gkargkavouzi et al., 2018; Herman, 2018; Lazarus and Folkman, 1984; Lohani et al., 2025b; Lohani et al., 2025a; Li and Monroe, 2019; Mathur and Kumari, 2013; Nisbet, 2009; Kelly et al., 2022; Kollmuss and Agyeman, 2002; Kovacs et al., 2024; Dunlap et al., 2000; Sanchez and Lafuente, 2010; Ojala, 2013; Park and Ha, 2012; Reser et al., 2012; Zaremba et al., 2022). (2) *Conflict* entails a reactive affect regulation approach that reflects maladaptive strategies that are a mismatch between intention and action around climate change (e.g., Ágoston et al., 2022; Clayton and Karazsia, 2020). (3) *Outcast* is also a reactive form that involves feeling alienated and maladaptive to manage climate change challenges (Carver et al., 1989; Choo, 2017; Folkman et al., 1986; Hornsey and Fielding, 2019; Tomassini et al., 2024; Zaremba et al., 2022). (4) *Spirituality-bodily practices* are stress-relief affect regulation approaches that include engagement in religious, spiritual, or relaxation practices for self-soothing (Bernard et al., 2022; Carver et al., 1989; Lohani et al., 2025b; Lohani et al., 2025c; Ojala, 2012b; White, 2018). (5) *Hope* is a proactive approach with strategies focusing on hope in scientists and community action to discover solutions to address climate change challenges (Janney et al., 2025; Lohani et al., 2025a; Lohani et al., 2025b; Ojala, 2023; Ojala, 2016; Ojala, 2015; Ojala, 2012b; Ojala, 2012a; Zaremba et al., 2022; Zummo et al., 2025). (6) *Apathy* is a reactive affect regulation approach that represents a combination of indifference, skepticism, and denial toward climate change (Carver et al., 1989; Davidson and Kecinski, 2021; Ding et al., 2011; Reser and Swim, 2011). (7) *Doom* reflects reactive affect regulation resulting in dysregulated states of feeling powerless and meaningless due to perceptions that the climate crisis is too big and unavoidable in being able to address (Zaremba et al., 2022; Reser and Swim, 2011; Reser et al., 2012). (8) *Overplaying* exaggerates or overemphasizes climate change in a way that undermines its significance. Specifically, the use of humor or mockery to neutralize or trivialize the emotional impact of the climate change crisis (Carroll-Monteil, 2023; Christie and Moore, 2005; Kaltenbacher and Drews, 2020; Simione and Gnagnarella, 2023; Skurka et al., 2018). (9) *Withdrawal* entails reactive affect regulation strategies aimed at avoiding dealing with one’s emotions around climate change, which may provide some relief from climate distress but would hinder climate action (Bloodhart et al., 2019; Carver et al., 1989; Davidson and Kecinski, 2021; Kovacs et al., 2024; Lazarus and Folkman, 1984; Lohani et al., 2025a; Norgaard, 2006; Zaremba et al., 2022).

**Table 5:** Description of the nine ECO-SHADOW factors.

ECO-SHADOW Factors	Definition	Category
Eco-consciousness	A proactive, problem-focused, and meaning-focused approach to managing emotions related to the climate crisis. Informed by a combination of personal values and responsibility, information seeking, and community-based climate action.	Proactive
Conflict	Strategies reflecting a contradiction between personal beliefs and actions (i.e., cognition dissonance), such as ambivalence, wishful thinking, procrastination, and self-blame. Represents a failure to effectively regulate behavior in line with personal beliefs and attitudes regarding climate change.	Reactive
Outcast	Efforts to manage feelings of isolation or alienation stemming from one’s personal beliefs, attitudes, and behavior toward climate change. Includes feelings of exclusion due to one’s concerns and actions (or lack thereof) about climate change. Can also reflect a reluctance to engage in open discussions about the climate crisis due to fear of being judged or misunderstood and is linked to engagement in maladaptive behaviors (e.g., substance abuse).	Reactive
Spirituality-bodily practice	Engagement in religious, spiritual, or relaxation practices for stress regulation.	Stress-relief
Hope	Acknowledgment of the climate change problem yet being rationally hopeful in scientists and community action to discover solutions to address climate change challenges.	Proactive
Apathy	An indifferent and skeptical approach toward climate change facts. May include emotional denial of climate change as a way of coping.	Reactive
Doom	Perceptions that climate change problems are too big and unavoidable, resulting in dysregulated states of feeling powerless and meaningless in being able to address the climate crisis.	Reactive
Overplay	Exaggeration or overemphasizing of climate change in a way that undermines its significance. Specifically, the use of humor or mockery to neutralize or trivialize the emotional impact of the climate change crisis.	Stress-relief
Withdrawal	Strategies aimed at avoiding dealing with one’s emotions around climate change (e.g., emotional avoidance, non-acceptance, and distraction). May provide some relief from climate distress but hinders climate action.	Reactive

### 3.4 Reliability and validity assessments

To examine reliability, the internal consistency of the scales was assessed through Cronbach’s alpha. Overall, the inventory showed excellent internal consistency (Cronbach’s *α* = 0.92), with an average inter-item correlation of *r* = 0.14 and a signal-to-noise ratio of 12.27. The standardized alpha (*α* = 0.92) and Guttman’s lambda-6 (*λ₆* = 0.96) further supported the reliability of the scale. Acceptable reliability based on Cronbach’s alpha was found for each of the nine scales. These reliability coefficients for each scale are presented in Table 3. Table 1 presents ECO-SHADOW items with factor loadings (FL, i.e., the value representing the item’s association with the underlying factor), communalities (*h^2^*, i.e., common variance for the item), and unique variance (*u^2^*, is the uniqueness score, 1-*h^2^*, i.e., variance only related to that item). Including all factor cross-loadings above 0.3 indicates a reasonable contribution of the included items to the underlying factor (Kline, 2015; Schreiber et al., 2006).

In order to examine validity through associations between the newly developed scale and established psychological constructs, SEM was employed to estimate latent correlations among the relevant variables. SEM allows for the estimation of correlations between latent variables while accounting for measurement error (Kline, 2015). Existing measures were utilized to examine validity. In the model, each construct—including the new scale and existing scales—was specified as a latent variable measured by its respective observed items. An estimated latent correlational analysis was conducted to examine convergent and divergent validity. A robust estimator was needed due to the non-normality violations, and accordingly, the model was estimated using the maximum likelihood robust (MLR) estimator in R, utilizing the *lavaan* package (Rosseel et al., 2017). Table 2 presents these validity comparisons. These latent correlations were interpreted to evaluate the strength and direction of associations between the new measure and theoretically related existing constructs. During convergent validity analysis, the preset criterion was the presence of a significant correlation between the existing measure and an ECO-SHADOW factor. In order to differentiate the ECO-SHADOW measure from unrelated measures, we conducted a discriminant validity analysis. The criterion for discriminant validity was a non-significant relationship between the existing measure and ECO-SHADOW factors.

## 4 Discussion

As climate change continues to worsen, understanding how people interface with their own psychological responses to this crisis is essential, as it has implications for their wellbeing and engagement with environmentally friendly efforts (Lohani et al., 2025b; Lohani et al., 2025a). We found that theoretical perspectives on affect regulation offer rich insights into diverse ways individuals may regulate their emotions (e.g., Aldao and Nolen-Hoeksema, 2012; Aldao et al., 2010; Heiy and Cheavens, 2014; Larsen, 2000; Lazarus and Folkman, 1984; Naragon-Gainey et al., 2017; Parkinson and Totterdell, 1999) regarding climate change. In this work, we extend existing literature on climate change and incorporate additional approaches from the general affect regulation literature to create an extensive list of strategies relevant to managing the climate crisis. Accordingly, we developed and validated a comprehensive inventory called ECO-SHADOW to assess the various ways university students regulate their emotional reactions to the realities of climate change. It captures the underlying structure of more than 60 affect regulation strategies identified in the context of climate change. These cognitive and behavioral regulation strategies converged into nine independent underlying factors: Eco-consciousness, Conflict, Outcast, Spiritual-bodily practices, Hope, Apathy, Doom, Overplay, and Withdrawal. Good reliability and validity metrics supported the generation of these nine factors, which help understand the underlying structure of affect regulation strategies relevant to the climate change context. Further work is needed to make the measure applicable beyond university students and generalize it to a broader and more diverse population.

In line with the proactive coping approach in relation to climate change (e.g., Homburg et al., 2007; Jovarauskaite and Böhm, 2020; Ojala, 2012b, 2013; Reser and Swim, 2011; Zaremba et al., 2022), *Eco-consciousness* was found to be a combination of cognitive and behavioral regulation targeting the wellbeing of all living things in the environment, including information seeking, community engagement, and mitigation (Lohani et al., 2025a; Lohani et al., 2025b). It incorporates cognitive strategies in which individuals aligning highly with this factor consider climate change’s causes and consequences and feel a moral responsibility to engage. At the same time, it has a significant component of pro-environmental behavioral strategies, such as volunteerism, planning and making climate-responsible decisions, advocating for environmental policies, and participating in personal and community sustainability projects. These behaviors necessitate a genuine concern and value for the welfare of both people and the planet. Notably, the Eco-consciousness factor includes a combination of problem- and meaning-focused strategies that may promote climate action, aligning with solution-focused young individuals who adopt these strategies (Rikner Martinsson and Ojala, 2024). It also correlated positively with other ECO-SHADOW factors representing strategies of conflict, withdrawal, hope, and being an outcast, suggesting that those who score high on the Eco-consciousness factor may engage in some additional strategies to manage their affect in response to climate change.

Notably, Eco-consciousness was linked negatively with the *Apathy* factor, suggesting that they oppose each other. In other words, those who engage in eco-consciousness do not exhibit an apathetic indifference toward climate change. This opposing relationship between eco-consciousness and apathy was also found in a recent study of community visitors to a climate change exhibit (Lohani et al., 2025b). Eco-consciousness was also correlated with existing measures of climate-related distress (Guan et al., 2024; Uppalapati et al., 2023) and with a “pro-ecological” environmental attitude (Dunlap et al., 2000). Additionally, Eco-consciousness was positively associated with experiences of negative affect, supporting prior arguments that such experiences can initiate climate action (Bright and Eames, 2021; Fielding and Hornsey, 2016; Howell, 2013; Syropoulos and Markowitz, 2022). This implies that a certain amount of distress around climate change issues is not harmful and is likely needed to initiate sustainability efforts (Lohani et al., 2025b). At the same time, eco-consciousness was linked to positive affect specific to climate change, indicating successful affect regulation and wellbeing despite caring for climate change. Overall, the Eco-consciousness factor represents potentially adaptive ways to regulate affect and engage in climate action effectively.

In contrast, Apathy was identified as an independent factor, representing a reactive regulation approach (e.g., Reser and Swim, 2011) that includes strategies that raise skepticism or foster indifference to the realities of climate change (Lohani et al., 2025b). The strategies underlying the Apathy factor were in line with previous work with young individuals, who reported a lack of care, denial, and deemphasis of the climate crisis (Bright and Eames, 2021; Ojala, 2012b). Supporting this theoretical perspective, Apathy was statistically negatively linked to Eco-consciousness. Unlike Eco-consciousness, Apathy was unrelated to experiences of positive affect when considering climate change. Notably, Apathy was conversely related to negative affect specific to climate change. Contrary to Eco-consciousness, Apathy was negatively related to pro-ecological environmental attitudes and positively related to “anti-ecological” environmental attitudes (i.e., dominant social paradigm, a less environmentally conscious worldview; Dunlap et al., 2000). On a related note, Apathy was statistically associated with a more disengaged, doubtful, or dismissive attitude toward climate change, as measured by a composite of SASSY (Chryst et al., 2018). This link was also replicated in a sample of community members (Lohani et al., 2025b). At the same time, Apathy was positively linked to experiences of spiritual-bodily practices and overplaying, signifying that apathetic individuals may. Given Apathy’s connections with anti-ecological environmental attitudes and beliefs, it appears to be a maladaptive approach in the context of climate change as it hinders climate action (e.g., Lohani et al., 2025b; Lohani et al., 2025a).

Unlike Apathy, *Withdrawal* includes catastrophic responses to climate change, and avoidance manages dysregulated feelings of overwhelm (Davidson and Kecinski, 2022). In line with this theoretical perspective, Withdrawal was statistically linked to climate change-related negative affect (*r* = 0.76), experiential avoidance (*r* = 0.57; Guan et al., 2024), and climate distress (*r* = 0.22; Uppalapati et al., 2023), reflecting the struggle individuals may face when trying to avoid dealing with climate change. As suggested by the universal worry item (#9.02 in Table 1), clustering with other avoidance items, despite efforts to avoid information and experiences related to the climate crisis, individuals may constantly worry about themselves and others being impacted by it. Withdrawal was also positively linked to pro-environmental attitudes (Dunlap et al., 2000) and views on global warming (Chryst et al., 2018). Accordingly, Withdrawal was positively correlated with other ECO-SHADOW factors, including Eco-consciousness, Conflict, Outcasted, Doom, and Overplay, and negatively associated with Apathy. Overall, Withdrawal may be a maladaptive approach to managing the climate crisis as it is ineffective in regulating affect and obstructs climate action.

In contrast to Withdrawal and Eco-consciousness, and similar to Apathy, *Doom* was not linked to experiences of positive affect. However, there are different reasons for the lack of positive affect among those high in Apathy versus Doom. While Apathy may stem from indifference and skepticism regarding climate change, those high in Doom are experiencing overwhelming climate distress without having effective ways to regulate the intense negativity and helplessness they feel. In other words, individuals scoring high on the Doom factor may be experiencing affective dysregulation due to climate change. Like Eco-consciousness and Withdrawal, Doom was positively correlated with climate-related distress, strong negative affect, and pro-environmental views (Chryst et al., 2018; Dunlap et al., 2000). Regarding other ECO-SHADOW factors, Doom was positively related to Conflict, Outcast, Overplay, and Withdrawal, and negatively associated with Spiritual-bodily practices. But Doom was unrelated to Eco-consciousness, Apathy, and Hope. Together, these findings suggest that Doom may be a maladaptive approach specific to regulating climate distress and is unlikely to promote meaningful sustainability efforts, and further work is needed to understand the relationship between different affect regulation families.

Another maladaptive affect regulation factor is *Outcast*, which entails feeling dysregulated while managing isolation stemming from one’s beliefs regarding climate change, leading to stonewalling or hesitation in sharing one’s true beliefs. Such extreme feelings can result in impaired behaviors and even substance abuse (Lohani et al., 2025a). In terms of other ECO-SHADOW factors, Outcast was positively linked to Conflict, Doom, Overplay, Eco-consciousness, and Withdrawal, and it was unrelated to Spiritual-bodily practices, Hope, or Apathy. Outcast differed from Eco-consciousness and Withdrawal in that it was unrelated to the pro- or anti-ecological paradigms. Future work should examine how feelings of social alienation can be experienced regardless of pro- or anti-ecological views on climate change. However, it did have weak positive links with climate-related avoidance, distress, negative affect, and supported pro-environment responses to SASSY (Chryst et al., 2018). The Outcast factor highlights the isolation many individuals may face due to their climate change responses relative to those around them and underscores the need to create a supportive space promoting open-minded conversations between individuals with diverse perspectives.

*Conflict* captures another type of dysregulation where one’s pro-environmental intentions do not align with actual behaviors, which can lead to negativity and dissociation. Unsurprisingly, Conflict was moderately correlated with negative affect and climate avoidance (Guan et al., 2024), and it has a weak relationship with climate distress (Uppalapati et al., 2023) and pro-environmental behaviors (Chryst et al., 2018; Dunlap et al., 2000). It was unrelated to Apathy, Hope, and Spiritual-bodily practices and positively related to Outcast, Doom, Overplay, Withdrawal, and Eco-consciousness. Unsuccessful efforts to regulate behaviors and act more sustainably in line with one’s wishes demonstrate that Conflict may be a maladaptive approach.

Conversely, the *Hope* factor was correlated with positive affect regarding climate change, implying its role in promoting adaptive regulation. These findings are in line with a recent study of museum-goers visiting a climate change exhibit, where visitors who were more hopeful about dealing with climate change had more intense positive responses (Lohani et al., 2025b). Indeed, hope plays a crucial role in motivating individuals to engage with climate change solutions (Brosch and Steg, 2021; Kelsey, 2020; Salas Reyes et al., 2021), with the potential to promote continued engagement (Morris et al., 2019) and influence policy support (Morris et al., 2019). *Rational hope*, in particular, could be a key approach, as it embodies an understanding of the magnitude of the problem while realistically considering solutions (Lohani et al., 2025b; Zummo et al., 2025). Along similar lines, Ojala (2023) called for centering *constructive hope* in climate education, conceptualizing it as a construct that includes both knowledge and affective processes. At the same time, Hope was positively correlated with Eco-consciousness, suggesting the importance of hope in climate action (Janney et al., 2025). Furthermore, Hope was also positively correlated with Spiritual-bodily practices, supporting past theoretical perspectives that highlight the inherent connection between religious climate engagement and the fostering of hope (White, 2018). Together, these findings imply that the Hope factor captures a distinct component of managing affective states that is also effective in promoting climate engagement, thereby suggesting that it is an adaptive approach (Janney et al., 2025; Lohani et al., 2025b; Lohani et al., 2025a; Ojala, 2012b; Ojala, 2012a; Zummo et al., 2025).

Regarding other stress-relief factors, *Spiritual-bodily practices* focus on engaging with religious, spiritual, and relaxation approaches. These strategies were negatively correlated with the Doom factor and positively linked to Hope and Apathy. At the same time, Spiritual-bodily practices did not correlate with existing climate change measures; a possible explanation could be that none of the items within the Spirituality-bodily practices explicitly refer to climate change. In future work, we will explicitly incorporate the climate change context in further iterations of this scale, and a suggested modification is included in the Appendix notes. Notably, the scale of the Spiritual-bodily practice still demonstrated good reliability and correlated with other climate-related affect regulation factors. This implies the importance of Spiritual-bodily practices in understanding regulatory efforts, as highlighted in past work (Bernard et al., 2022; Pihkala, 2022b). Indeed, a recent study found religion, exercise, yoga, meditation, and relaxation as an effective approach to managing climate distress (Lohani et al., 2025a; Lohani et al., 2025b), implying the significance of such stress-relief practices in regulating climate distress. These are the reasons to still keep the Spiritual-bodily practices scale in ECO-SHADOW because it has good validity and theoretical relevance to climate change. Given that this factor correlated with both Hope and Apathy and its lack of association with existing pro-environmental measures, it remains to be determined what may lead spiritual-bodily practices to be an adaptive versus a maladaptive approach in the climate change context.

*Overplay* focuses on using mockery or humor to deal with climate change challenges and is similar to Apathy in that it also has a significant negative, weak relationship with negative affect and pro-environmental beliefs and attitudes (Chryst et al., 2018; Dunlap et al., 2000). However, unlike Apathy, overplay is unrelated to Eco-consciousness, but still shows a weak relationship with Conflict, Outcast, Doom, Withdrawal, and Apathy. Notably, the Barratt Impulsiveness Scale (Barratt, 1975) was selected to test discriminant validity, and all ECO-SHADOW factors, as expected, were unrelated to the three scales: Attention, Motor, and Non-planning, with one exception. Overplay was unrelated to the Attention and Motor scales but was found to have a weak (*r* = 0.09, *p* = 0.03) association with Non-planning Impulsivity, which captures an inability to plan ahead for the future. It is understandable that the Overplay factor was linked to non-planning impulsivity, as those who score higher on this factor may be adopting humor and mockery to manage their impulsive nature. Given that the other two scales (Attention and Motor) did not relate to Overplay, indicating discriminant validity, and that Overplay showed convergent validity with other existing measures, we kept it as one of the factors because of its relevance in managing challenging climate change-related situations (Carroll-Monteil, 2023; Kaltenbacher and Drews, 2020). Overplay is an important regulatory approach to capture because it may be maladaptive; as warned by recent work, it may temporarily reduce negativity, but work against the promotion of pro-climate behaviors by decreasing the perceived risk of the climate crisis (Skurka et al., 2018). In general, further work is needed to understand the effectiveness of affect regulation strategies in the context of climate change, as it may depend on the goals of an individual (e.g., experiencing wellbeing) and the impact it may have on the trajectory of climate change (e.g., climate action).

## 5 Implications

### 5.1 Contributions and outstanding questions to promote sustainable climate action

The ECO-SHADOW inventory provides the underlying structure of wide-ranging affect regulation strategies that are implemented by young adults to manage their responses in the context of climate change. This work provides to-date the most exhaustive list of cognitive and behavioral approaches adopted by college students, and further work is needed to extend the scope to a more diverse, generalizable population. As captured by factors such as Doom, Outcast, Withdrawal, and Conflict, the widespread perception of climate change as an imminent and overwhelming threat can create despair and hopelessness. This perception, characterized by a sense of “doom and gloom,” can hinder individuals and communities from enacting meaningful responses to address the climate change crisis. Research indicates that negative emotions associated with climate change significantly influence individuals’ readiness to adopt pro-climate behaviors (Armstrong et al., 2025; Bright and Eames, 2021; Brosch and Steg, 2021; Davidson and Kecinski, 2022; Kovacs et al., 2024; van der Linden, 2014). In climate scholarship, negative affective states like doom, gloom, and hopelessness are recognized as significant obstacles to supporting pro-climate action and policy (Brosch and Steg, 2021; Davidson and Kecinski, 2022; Kovacs et al., 2024; van der Linden, 2014). In contrast, the Eco-consciousness factor encompasses 18 affect regulation approaches that involve pro-environmental cognitions and behaviors. These strategies need further consideration as they may maximize environmental action while minimizing personal harm and distress. Thus, an important contribution of the current ECO-SHADOW inventory is that it serves as a helpful tool for identifying individual differences in affect regulation approaches and their implications for sustainability efforts. At the same time, much remains to be learned about effective interventions to support a diverse group of individuals in transforming doom and gloom into proactive and productive sustainable actions.

Another critical question facing the field is what constitutes adaptive regulation in the context of climate change. Several theoretical perspectives have argued that experiences of negative affect, to some extent, may be necessary for action (Hill and Lohani, n.d.; Ortner et al., 2018; Tamir, 2016). The current work, along with past literature, supports this theoretical perspective in the context of climate change, demonstrating that emotional responses to the climate crisis are indeed adaptive (Cunsolo et al., 2020; Lohani et al., 2025b; Verplanken and Roy, 2013) rather than maladaptive (Budziszewska and Kałwak, 2022). Furthermore, evidence-based interventions are needed to channel climate-related distress into adaptive responses (Crandon et al., 2024). It is important to gain an understanding of who is more susceptible to climate distress (such as those individuals scoring high on Withdrawal, Outcast, Conflict, and Doom scales) and what can be done to mitigate it (Searle and Gow, 2010). At the same time, if personal affect regulation efforts are solely directed at feeling better personally—such as by employing apathy and humor —without regard for sustainability efforts, then such efforts should be considered maladaptive. Thus, further work is needed to continue testing adaptive ways of affect regulation (such as eco-consciousness) that support an optimal level of well being while also fostering climate engagement and action. Further work is also needed to effectively communicate and engage the community in sustainability efforts (Janney et al., 2024; Nabi et al., 2018).

### 5.2 Limitations and future directions

The ECO-SHADOW inventory provides the most comprehensive measure of affect regulation strategies for managing one’s reactions to the climate change crisis; however, this work should be interpreted with the following limitations in mind. In our efforts to be comprehensive, we have attempted to include as many relevant strategies as possible. This led to the first limitation: the inventory is currently long. Ongoing iterative data collection efforts are targeted to make the measure shorter. Being mindful of time, a researcher can adopt a few of the nine independent scales (such as Eco-consciousness and Apathy) to capture constructs of interest without needing to administer them all. Second, we need a more representative sample to improve its generalizability. Ongoing efforts are focused on collecting data from a diverse sample that spans the full range from feeling alarmed to dismissive perspectives regarding climate change. On a related note, future work could involve cross-validation of the factor structure on an independent sample using Confirmatory Factor Analysis (CFA), which would further strengthen support for the ECO-SHADOW inventory. Sample size limitations restricted an examination of measurement invariance testing across groups (e.g., political ideology, age, etc.) as well as higher-order factor analysis, which is an important future direction for this work. Given the cross-sectional nature of current findings, future longitudinal and experimental follow-up studies (Lohani and Blodgett, 2025) are needed to understand the causal relationships and strengthen the generalizability of the findings.

Third, it is possible that people may adopt multiple strategies simultaneously (Ford et al., 2019). Note that the factors are independent, but they may still correlate. The sub-groups that utilize multiple strategies across factors remain to be understood. Fourth, although it is challenging to capture all specific environment-related behaviors, and we did not ask specific ones, some researchers have identified specific strategies that individuals may adopt (e.g., pro-environment behavior, power conservation, ecologically aware consumer behavior, water conservation, rationale automobile use, ecological waste management; Markle, 2013). Future research should examine a comprehensive measure of climate action as correlates and outcomes of cognitive and behavioral affect regulation. Finally, further work is needed to understand the moderators that play a role in affect regulation processes, including personal (e.g., identity, political beliefs), environmental factors (e.g., experiencing direct impacts of climate change), and community factors (e.g., cultural norms; Crandon et al., 2024; Reser and Swim, 2011).

## 6 Conclusion

How one regulates their emotions in the context of climate crisis matters not only for their personal wellbeing but also for the wellbeing of the planet. The ECO-SHADOW inventory is a reliable, valid, and thus far the most exhaustive measure of the wide-ranging cognitive and behavioral techniques young adults may adopt to manage climate change. Although there is meaningful overlap between these scales, they are theoretically and statistically distinct from one another, allowing researchers to assess individual factors depending on the needs of the research project. This inventory provides a means to capture individual differences in affect regulation approaches regarding climate change, with some being more adaptive (i.e., eco-consciousness and hope) to climate action, while others are less so (e.g., apathy, withdrawal, doom, or overplay). It is possible that individuals who score high on Doom, Withdrawal, Conflict, and Overplay scales may need emotional support to improve their personal wellbeing and find constructive ways to facilitate pro-environment actions. In contrast, those who may be highly apathetic and isolated may require educational programs to motivate them to adopt an environmentally friendly perspective. Further work is required to test these suggestions from the current research and identify effective ways to manage emotions that will maintain wellbeing and maximize climate-friendly behaviors. We hope that the ECO-SHADOW inventory inspires future research promoting effective affect regulation and its connections to sustainable climate action.

## Data Availability

The raw data supporting the conclusions of this article will be made available by the authors, upon reasonable request.

## References

[ref1] Ágoston C. Csaba B. Nagy B. Kőváry Z. Dúll A. Rácz J.. (2022). Identifying types of eco-anxiety, eco-guilt, eco-grief, and eco-coping in a climate-sensitive population: a qualitative study. Int J Environ Res Public Health 19:2461. doi: 10.3390/ijerph19042461, PMID: 35206648 PMC8875433

[ref2] Ahmed S. P. Bittencourt-Hewitt A. Sebastian C. L. (2015). Neurocognitive bases of emotion regulation development in adolescence. Dev Cogn Neurosci 15, 11–25. doi: 10.1016/j.dcn.2015.07.006, PMID: 26340451 PMC6989808

[ref3] Aldao A. Nolen-Hoeksema S. (2012). When are adaptive strategies most predictive of psychopathology? J Abnorm Psychol 121, 276–281. doi: 10.1037/a0023598, PMID: 21553934

[ref4] Aldao A. Nolen-Hoeksema S. Schweizer S. (2010). Emotion-regulation strategies across psychopathology: a meta-analytic review. Clin Psychol Rev 30, 217–237. doi: 10.1016/j.cpr.2009.11.004, PMID: 20015584

[ref5] Anderson (2012). “New ecological paradigm (NEP) scale” in Berkshire encyclopedia of sustainability. Eds. I. Spellerberg, D. S. Fogel, B. Harrington, S. E. Fredericks. (Great Barrington, MA: Berkshire Publishing Group).

[ref6] Armstrong H. E. Parker P. C. Ortner C. N. M. (2025). Emotions and climate change: the role of emotion regulation in climate action. Emotion, 1–13. doi: 10.1037/emo0001546 40402609

[ref7] Barratt E. S. (1975). Barratt impulsiveness scale: ETS m 1975. Galveston, Texas: Barratt-Psychiatry Medical Branch, University of Texas.

[ref8] Bartlett M. S. (1954). A note on the multiplying factors for various χ^2^ approximations. J R Stat Soc Ser B Methodol 16, 296–298. doi: 10.1111/j.2517-6161.1954.tb00174.x

[ref9] Bernard P. Chevance G. Kingsbury C. Gadais T. Dancause K. Villarino R.. (2022). Climate change: the next game changer for sport and exercise psychology. Ger J Exerc Sport Res 54, 6–11. doi: 10.1007/s12662-022-00819-w PMC9098786 40479168

[ref10] Berto R. (2014). The role of nature in coping with psycho-physiological stress: a literature review on restorativeness. Behav Sci 4, 394–409. doi: 10.3390/bs4040394, PMID: 25431444 PMC4287696

[ref11] Bloodhart B. Swim J. K. Dicicco E. (2019). “Be worried, be VERY worried:” preferences for and impacts of negative emotional climate change communication. Front Commun 3, 1–15. doi: 10.3389/fcomm.2018.00063

[ref12] Böhm G. Pfister H.-R. Doran R. Ogunbode C. A. Poortinga W. Tvinnereim E.. (2023). Emotional reactions to climate change: a comparison across France, Germany, Norway, and the United Kingdom. Front Psychol 14, 1–23. doi: 10.3389/fpsyg.2023.1139133, PMID: 37484093 PMC10358841

[ref13] Bond F. W. Hayes S. C. Baer R. A. Carpenter K. M. Guenole N. Orcutt H. K.. (2011). Preliminary psychometric properties of the acceptance and action questionnaire–II: a revised measure of psychological inflexibility and experiential avoidance. Behav Ther 42, 676–688. doi: 10.1016/j.beth.2011.03.007, PMID: 22035996

[ref14] Bord R. J. O'connor R. E. Fisher A. (2000). In what sense does the public need to understand global climate change? Public Underst Sci 9, 205–218. doi: 10.1088/0963-6625/9/3/301

[ref15] Bradley G. L. Reser J. P. Glendon A. I. Ellul M. C. (2014). “Distress and coping in response to climate change” in Stress and anxiety: Applications to social and environmental threats, psychological well-being, occupational challenges, and developmental psychology climate change. Eds. K. Kaniasty, P. Buchwald, S. Howard, A. Kathleen. (Berlin, Germany:Logos Verlag Berlin). 33–42.

[ref16] Bright M. L. Eames C. (2021). From apathy through anxiety to action: emotions as motivators for youth climate strike leaders. Aust J Environ Educ 38, 1–13. doi: 10.1017/aee.2021.22

[ref17] Bronfman N. C. Cisternas P. C. López-Vázquez E. De la Maza C. Oyanedel J. C. (2015). Understanding attitudes and pro-environmental behaviors in a Chilean community. Sustainability 7, 14133–14152. doi: 10.3390/su71014133

[ref18] Brosch T. Steg L. (2021). Leveraging emotion for sustainable action. One Earth 4, 1693–1703. doi: 10.1016/j.oneear.2021.11.006

[ref19] Brown T. A. (2015). Confirmatory factor analysis for applied research. New York, NY: Guilford Press.

[ref20] Budziszewska M. Kałwak W. (2022). Climate depression. Critical analysis of the concept. Psychiatr Pol 56, 171–182. doi: 10.12740/PP/127900, PMID: 35569156

[ref21] Carroll-Monteil E. (2023). Is climate change a laughing matter? Environ Educ Res 29, 569–591. doi: 10.1080/13504622.2022.2113764

[ref22] Carver C. (2020). “Coping” in Encyclopedia of behavioral medicine. Editor. M. D. Gellman. (Cham: Springer), 550–554.

[ref23] Carver C. S. Scheier M. F. Weintraub J. K. (1989). Assessing coping strategies: a theoretically based approach. J Pers Soc Psychol 56, 267–283. 2926629 10.1037//0022-3514.56.2.267

[ref24] Choo C. W. (2017). Seeking and avoiding information in a risky world. Inf Res 22:765.

[ref25] Christie W. Moore C. (2005). The impact of humor on patients with Cancer. Clin J Oncol Nurs 9, 211–218. doi: 10.1188/05.CJON.211-218 15853164

[ref26] Chryst B. Marlon J. Van Der Linden S. Leiserowitz A. Maibach E. Roser-Renouf C. (2018). Global warming’s “six Americas short survey”: audience segmentation of climate change views using a four question instrument. Env Communication 12, 1109–1122. doi: 10.1080/17524032.2018.1508047

[ref27] Clayton S. Karazsia B. T. (2020). Development and validation of a measure of climate change anxiety. J Environ Psychol 69:101434. doi: 10.1016/j.jenvp.2020.101434, PMID: 40698065

[ref28] Costello A. B. Osborne J. W. (2005). Best practices in exploratory factor analysis: four recommendations for getting the most from your analysis. Pract Assess Res Eval 10, 1–9. doi: 10.7275/jyj1-4868

[ref29] Coutlee C. G. Politzer C. S. Hoyle R. H. Huettel S. A. (2014). An abbreviated impulsiveness scale constructed through confirmatory factor analysis of the Barratt impulsiveness scale version 11. Arch Sci Psychol 2, 1–12. doi: 10.1037/arc0000005, PMID: 26258000 PMC4527550

[ref30] Crandon T. J. Scott J. G. Charlson F. J. Thomas H. J. (2024). A theoretical model of climate anxiety and coping. Discov Psychol 4:94. doi: 10.1007/s44202-024-00212-8

[ref31] Cunsolo A. Harper S. L. Minor K. Hayes K. Williams K. G. Howard C. (2020). Ecological grief and anxiety: the start of a healthy response to climate change? Lancet Planetary Health 4, e261–e263. doi: 10.1016/S2542-5196(20)30144-3, PMID: 32681892

[ref32] Davidson D. J. Kecinski M. (2021). Emotional pathways to climate change responses. WIREs Clim Change 13, 1–19. doi: 10.1002/wcc.751

[ref33] Davidson D. J. Kecinski M. (2022). Emotional pathways to climate change responses. Wiley Interdiscip Rev Clim Chang 13:e751.

[ref34] De Groot J. I. Steg L. (2007). Value orientations and environmental beliefs in five countries: validity of an instrument to measure egoistic, altruistic and biospheric value orientations. J Cross-Cult Psychol 38, 318–332. doi: 10.1177/0022022107300278

[ref35] Dietz T. Dan A. Shwom R. (2007). Support for climate change policy: social psychological and social structural influences. Rural Sociol 72, 185–214. doi: 10.1526/003601107781170026

[ref36] Ding D. Maibach E. W. Zhao X. Roser-Renouf C. Leiserowitz A. (2011). Support for climate policy and societal action are linked to perceptions about scientific agreement. Nat Clim Chang 1, 462–466. doi: 10.1038/nclimate1295

[ref37] Duchi L. Lombardi D. Paas F. Loyens S. M. M. (2020). How a growth mindset can change the climate: the power of implicit beliefs in influencing people’s view and action. J Environ Psychol 70:101461. doi: 10.1016/j.jenvp.2020.101461, PMID: 40698065

[ref38] Dunlap R. E. Van Liere K. D. (1978). The “new environmental paradigm”. J Environ Educ 9, 10–19. doi: 10.1080/00958964.1978.10801875

[ref39] Dunlap R. E. Van Liere K. D. Mertig A. G. Jones R. E. (2000). New trends in measuring environmental attitudes: measuring endorsement of the new ecological paradigm: a revised NEP scale. J Soc Issues 56, 425–442. doi: 10.1111/0022-4537.00176

[ref40] Epskamp S. Stuber S. Nak J. Veenman M. Jorgensen T. D. (2022) Package ‘semPlot’ Available online at: https://cran.r-project.org/web/packages/semPlot/semPlot.pdf

[ref41] Fabrigar L. R. Wegener D. T. MacCallum R. C. Strahan E. J. (1999). Evaluating the use of exploratory factor analysis in psychological research. Psychol Methods 4, 272–299. doi: 10.1037/1082-989X.4.3.272

[ref42] Fielding K. S. Hornsey M. J. (2016). A social identity analysis of climate change and environmental attitudes and behaviors: insights and opportunities. Front Psychol 7, 1–12. doi: 10.3389/fpsyg.2016.00121, PMID: 26903924 PMC4749709

[ref43] Folkman S. Lazarus R. S. Dunkel-Schetter C. DeLongis A. Gruen R. (1986). The dynamics of a stressful encounter: cognitive appraisal, coping and encounter outcomes. J Pers Soc Psychol 50, 992–1003. 3712234 10.1037//0022-3514.50.5.992

[ref44] Ford B. Q. Gross J. J. Gruber J. (2019). Broadening our field of view: the role of emotion polyregulation. Emot Rev 11, 197–208. doi: 10.1177/1754073919850314

[ref45] Garnefski N. Kraaij V. (2006). Cognitive emotion regulation questionnaire: development of a short 18-item version (CERQ-short). Personal Individ Differ 41, 1045–1053. doi: 10.1016/j.paid.2006.04.010

[ref46] Gkargkavouzi A. Paraskevopoulos S. Matsiori S. (2018). Who cares about the environment? J Hum Behav Soc Environ 28, 746–757. doi: 10.1080/10911359.2018.1458679

[ref47] Grommisch G. Koval P. Hinton J. D. Gleeson J. Hollenstein T. Kuppens P.. (2020). Modeling individual differences in emotion regulation repertoire in daily life with multilevel latent profile analysis. Emotion 20, 1462–1474. doi: 10.1037/emo0000669, PMID: 31478725

[ref48] Gross J. J. (1998). The emerging field of emotion regulation: an integrative review. Rev Gen Psychol 2, 271–299. doi: 10.1037/1089-2680.2.3.271

[ref49] Guan JYN Dutcher E Goldin P. (2024) The role of mindfulness in moderating climate distress during wildfire season. 3, 0000524. doi: 10.1371/journal.pclm.0000524

[ref51] Heiy J. E. Cheavens J. S. (2014). Back to basics: a naturalistic assessment of the experience and regulation of emotion. Emotion 14, 878–891. doi: 10.1037/a0037231, PMID: 24999913

[ref52] Herman B. C. (2018). Students' environmental NOS views, compassion, intent, and action: impact of place-based socioscientific issues instruction. J Res Sci Teach 55, 600–638. doi: 10.1002/tea.21433

[ref53] Hill P. L. Lohani M. (n.d.). Comfortably distressed in the pursuit of purpose: how negative emotions and their regulation can shape purpose development. New Ideas Psychol.

[ref54] Hine D. W. Phillips W. J. Cooksey R. Reser J. P. Nunn P. Marks A. D. G.. (2016). Preaching to different choirs: how to motivate dismissive, uncommitted, and alarmed audiences to adapt to climate change? Glob Environ Chang 36, 1–11. doi: 10.1016/j.gloenvcha.2015.11.002

[ref55] Homburg A. Stolberg A. Wagner U. (2007). Coping with global environmental problems: development and first validation of scales. Environ Behav 39, 754–778. doi: 10.1177/0013916506297215

[ref56] Hornsey M. J. Fielding K. S. (2019). Understanding (and reducing) inaction on climate change. Soc Issues Policy Rev 14, 3–35. doi: 10.1111/sipr.12058

[ref57] Howell R. A. (2013). It’s not (just) “the environment, stupid!” values, motivations, and routes to engagement of people adopting lower-carbon lifestyles. Glob Environ Chang 23, 281–290. doi: 10.1016/j.gloenvcha.2012.10.015

[ref58] Hu L. T. Bentler P. M. (1999). Cutoff criteria for fit indexes in covariance structure analysis: conventional criteria versus new alternatives. Struct Equ Model 6, 1–55. doi: 10.1080/10705519909540118

[ref59] Janney B. A. Zummo L. Lohani M. (2024). Shifting the desired outcome from climate literacy to climate agency: education that empowers civic leaders. Interdis J Environ Sci Educ 20:e2412. doi: 10.29333/ijese/14657

[ref60] Janney B. Zummo L. Lohani M. Sanchez-Torres S. Bertuzzi D. (2025). Fostering Hope: exploring climate change learning in an informal museum setting. Visitor Stud 1–18, 1–18. doi: 10.1080/10645578.2024.2446135, PMID: 40640861

[ref61] Jones C. Lucas C. (2023). “Listen to me!”: young people’s experiences of talking about emotional impacts of climate change. Glob Environ Chang 83:102744. doi: 10.1016/j.gloenvcha.2023.102744

[ref62] Jovarauskaite L. Böhm G. (2020). The emotional engagement of climate experts is related to their climate change perceptions and coping strategies. J Risk Res 24, 941–957. doi: 10.1080/13669877.2020.1779785

[ref63] Kaiser H. F. (1974). An index of factorial simplicity. Psychometrika 39, 31–36.

[ref64] Kalokerinos E. K. Tamir M. Kuppens P. (2017). Instrumental motives in negative emotion regulation in daily life: frequency, consistency, and predictors. Emotion 17, 648–657. doi: 10.1037/emo0000269, PMID: 27991816

[ref65] Kaltenbacher M. Drews S. (2020). An inconvenient joke? A review of humor in climate change communication. Environ Commun 14, 717–729. doi: 10.1080/17524032.2020.1756888

[ref66] Kelly R. Elsler L. G. Polejack A. van der Linden S. Tönnesson K. Schoedinger S. E.. (2022). Empowering young people with climate and ocean science: five strategies for adults to consider. One Earth 5, 861–874. doi: 10.1016/j.oneear.2022.07.007

[ref67] Kelsey E. (2020). Hope matters: Why changing the way we think is critical to solving the environmental crisis. Vancouver, British Columbia, Canada: Greystone Books Ltd.

[ref68] Kline R. B. (2015). Principles and practice of structural equation modeling. New York, NY: Guilford Press.

[ref69] Kollmuss A. Agyeman J. (2002). Mind the gap: why do people act environmentally and what are the barriers to pro-environmental behavior? Environ Educ Res 8, 239–260. doi: 10.1080/13504620220145401

[ref70] Kovacs L. N. Jordan G. Berglund F. Holden B. Niehoff E. Pohl F.. (2024). Acting as we feel: which emotional responses to the climate crisis motivate climate action. J Environ Psychol 96:102327. doi: 10.1016/j.jenvp.2024.102327

[ref71] Kroenke K. Spitzer R. L. Williams J. B. (2003). The patient health questionnaire-2: validity of a two-item depression screener. Med Care 41, 1284–1292. doi: 10.1097/01.MLR.0000093487.78664.3C, PMID: 14583691

[ref72] Kroenke K. Spitzer R. L. Williams J. B. W. Monahan P. O. Löwe B. (2007). Anxiety disorders in primary care: prevalence, impairment, comorbidity, and detection. Ann Intern Med 146, 317–325. doi: 10.7326/0003-4819-146-5-200703060-00004, PMID: 17339617

[ref73] Larsen R. J. (2000). Toward a science of mood regulation. Psychol Inq 11, 129–141. doi: 10.1207/S15327965PLI1103_01

[ref74] Lazarus R. S. Folkman S. (1984). Stress, appraisal and coping. New York: Springer.

[ref75] Leiserowitz A. Thaker J. Feinberg G. Cooper D. (2013). Globalav warming’s six Indias. Yale University. New Haven, CT: Yale Project on Climate Change Communication.

[ref76] Li C. J. Monroe M. C. (2019). Exploring the essential psychological factors in fostering hope concerning climate change. Environ Educ Res 25, 936–954. doi: 10.1080/13504622.2017.1367916

[ref78] Lohani M. Blodgett G. (2025). Innovative and ecological: integrating ecological momentary assessment into environmental science research. Front Psychol 16:1557055. doi: 10.3389/fpsyg.2025.1557055, PMID: 40302913 PMC12037503

[ref79] Lohani M Cachelin A. Banerjee D. Brunelle A. (2025a). Student Responses to the Climate Crisis: Managing Distress and Exploring Support Systems. Int J SUST Higher Ed.

[ref80] Lohani M. Dutton S. Elsey J. S. (2022). A day in the life of a college student during the COVID-19 pandemic: an experience sampling approach to emotion regulation. Appl Psychol Health Well Being 14, 1333–1352. doi: 10.1111/aphw.12337, PMID: 35023310

[ref81] Lohani M. Elsey J. S. Dutton S. Zummo L. (2025c). Climate Change is Linked to and Daily Wellbeing: The Role of Environmental, Governmental, and Commute-Related Stressors. 10.1080/28324765.2025.2539201 PMC12536717 41262958

[ref82] Lohani M. Zummo L. Brunelle A. Shah J. Cachelin A. Yeo S.. (n.d.-a). Together, we learn and make a difference: emotion regulation strategies among climate science students.

[ref83] Lohani M. Zummo L. Janney B. A. Blodgett G. R. (n.d.-b). Managing climate emotions: How emotion regulation is integral to climate engagement.

[ref84] Lohani M. McElvaine K. Payne B. Mitcheom K. Britton W. (2020). A longitudinal training study to delineate the specific causal effects of open monitoring versus focused attention techniques on emotional health. Complement Ther Med 53:102525. doi: 10.1016/j.ctim.2020.102525, PMID: 33066868

[ref85] Lohani M. Pfund G. N. Bono T. J. Hill P. L. (2023). Starting school with purpose: self-regulatory strategies of first-semester university students. Appl Psychol Health Well Being 15, 723–739. doi: 10.1111/aphw.12407, PMID: 36217594 PMC10083189

[ref86] Lohani M. Zummo L. Janney B. Giron J. (2025b). Exploring emotional reactions and regulation strategies in climate change contexts: insights from a museum exhibit. J Mus Educ, 1–15. doi: 10.1080/10598650.2025.2494873

[ref87] Maibach E. W. Leiserowitz A. Roser-Renouf C. Mertz C. K. (2011). Identifying like-minded audiences for global warming public engagement campaigns: an audience segmentation analysis and tool development. PLoS One 6:e17571. doi: 10.1371/journal.pone.0017571, PMID: 21423743 PMC3053362

[ref88] Markle G. L. (2013). Pro-environmental behavior: does it matter how it’s measured? Development and validation of the pro-environmental behavior scale (PEBS). Hum Ecol 41, 905–914. doi: 10.1007/s10745-013-9614-8

[ref89] Mathur V. Kumari K. M. (2013). Environmental consciousness: an indicator of higher consciousness. Int J Sci Res Publ 3, 1–5.

[ref91] McMichael A. J. Lindgren E. (2011). Climate change: present and future risks to health, and necessary responses. J Intern Med 270, 401–413. doi: 10.1111/j.1365-2796.2011.02415.x, PMID: 21682780

[ref92] Medland H. De France K. Hollenstein T. Mussoff D. Koval P. (2020). Regulating emotion systems in everyday life. Eur J Psychol Assess 36, 437–446. doi: 10.1027/1015-5759/a000595

[ref93] Metag J. Füchslin T. Schäfer M. S. (2017). Global warming’s five Germanys: a typology of Germans’ views on climate change and patterns of media use and information. Public Underst Sci 26, 434–451. doi: 10.1177/0963662515592558, PMID: 26142148

[ref94] Morris B. S. Chrysochou P. Christensen J. D. Orquin J. L. Barraza J. Zak P. J.. (2019). Stories vs. facts: triggering emotion and action-taking on climate change. Clim Chang 154, 19–36. doi: 10.1007/s10584-019-02425-6, PMID: 40693173

[ref95] Nabi R. L. Gustafson A. Jensen R. (2018). Framing climate change: exploring the role of emotion in generating advocacy behavior. Sci Commun 40, 442–468. doi: 10.1177/1075547018776019

[ref96] Naragon-Gainey K. McMahon T. P. Chacko T. P. (2017). The structure of common emotion regulation strategies: a Meta-analytic examination. Psychol Bull 143, 384–427. doi: 10.1037/bul0000093, PMID: 28301202

[ref97] Nisbet M. C. (2009). “Framing science: a new paradigm in public engagement” in Communicating science. Eds. L. Kahlor, P. Stout. (New York, NY: Routledge), 54–81.

[ref98] Nisbet E. K. Zelenski J. M. (2013). The NR-6: a new brief measure of nature relatedness. Front Psychol 4:813. doi: 10.3389/fpsyg.2013.00813, PMID: 24198806 PMC3814587

[ref99] Norgaard K. M. (2006). “People want to protect themselves a little bit”: emotions, denial, and social movement nonparticipation*. Sociol Inq 76, 372–396. doi: 10.1111/j.1475-682x.2006.00160.x

[ref100] O’Connor R. E. Bord R. J. Yarnal B. Wiefek N. (2002). Who wants to reduce greenhouse gas emissions? Soc Sci Q 83, 1–17. doi: 10.1111/1540-6237.00067

[ref101] Ogunbode C. A. Böhm G. Capstick S. B. Demski C. Spence A. Tausch N. (2019). The resilience paradox: flooding experience, coping and climate change mitigation intentions. Clim Pol 19, 703–715. doi: 10.1080/14693062.2018.1560242

[ref102] Ogunbode C. A. Pallesen S. Böhm G. Doran R. Bhullar N. Aquino S.. (2021). Negative emotions about climate change are related to insomnia symptoms and mental health: cross-sectional evidence from 25 countries. Curr Psychol 42, 845–854. doi: 10.1007/s12144-021-01385-4

[ref103] Ojala M. (2012a). Hope and climate change: the importance of hope for environmental engagement among young people. Environ Educ Res 18, 625–642. doi: 10.1080/13504622.2011.637157

[ref104] Ojala M. (2012b). Regulating worry, promoting hope: how do children, adolescents, and young adults cope with climate change? Int J Environ Sci Educ 7, 537–561.

[ref105] Ojala M. (2013). Coping with climate change among adolescents: implications for subjective well-being and environmental engagement. Sustainability 5, 2191–2209. doi: 10.3390/su5052191

[ref106] Ojala M. (2015). Hope in the face of climate change: associations with environmental engagement and student perceptions of teachers’ emotion communication style and future orientation. J Environ Educ 46, 133–148. doi: 10.1080/00958964.2015.1021662

[ref107] Ojala M. (2016). Facing anxiety in climate change education: from therapeutic practice to hopeful transgressive learning. Can J Environ Educ 21, 41–56.

[ref108] Ojala M. (2023). Hope and climate-change engagement from a psychological perspective. Curr Opin Psychol 49:101514. doi: 10.1016/j.copsyc.2022.101514, PMID: 36502586

[ref109] O'Neill S. Nicholson-Cole S. (2009). “Fear won't do it” promoting positive engagement with climate change through visual and iconic representations. Sci Commun 30, 355–379. doi: 10.1177/1075547008329201

[ref110] Ortner C. N. Corno D. Fung T. Y. Rapinda K. (2018). The roles of hedonic and eudaimonic motives in emotion regulation. Pers Individ Differ 120, 209–212. doi: 10.1016/j.paid.2017.09.006

[ref111] Park J. Ha S. (2012). Understanding pro-environmental behavior. Int J Retail Distrib Manag 40, 388–403. doi: 10.1108/09590551211222367

[ref112] Parkinson B. Totterdell P. (1999). Classifying affect-regulation strategies. Cognit Emot 13, 277–303. doi: 10.1080/026999399379285

[ref113] Pihkala P. (2022a). Toward a taxonomy of climate emotions. Front Climate 3, 1–22. doi: 10.3389/fclim.2021.738154

[ref114] Pihkala P. (2022b). Eco-anxiety and pastoral care: theoretical considerations and practical suggestions. Religion 13:192. doi: 10.3390/rel13030192

[ref115] Radakovic R. McGrory S. Chandran S. Swingler R. Pal S. Stephenson L.. (2020). The brief dimensional apathy scale: a short clinical assessment of apathy. Clin Neuropsychol 34, 423–435. doi: 10.1080/13854046.2019.1621382 31154933

[ref116] Raes F. Pommier E. Neff K. D. Van Gucht D. (2011). Construction and factorial validation of a short form of the self-compassion scale. Clin Psychol Psychother 18, 250–255. doi: 10.1002/cpp.702, PMID: 21584907

[ref117] Reser J. P. Bradley G. L. Glendon A. I. Ellul M. C. Callaghan R. (2012). Public risk perceptions, understandings and responses to climate change in Australia and Great Britain, Gold Coast, Australia: National Climate Change Adaptation Research Facility. 298.

[ref118] Reser J. P. Swim J. K. (2011). Adapting to and coping with the threat and impacts of climate change. Am Psychol 66, 277–289. doi: 10.1037/a0023412 21553953

[ref119] Revelle W. Revelle M. W. (2015). Package ‘psych’. Comprehensive R Archive Network 337, 161–165.

[ref120] Rikner Martinsson A. Ojala M. (2024). Patterns of climate-change coping among late adolescents: differences in emotions concerning the future, moral responsibility, and climate-change engagement. Clim Chang 177:125. doi: 10.1007/s10584-024-03778-3

[ref121] Roelvink G. Zolkos M. (2011). Climate change as experience of affect. Angelaki 16, 43–57. doi: 10.1080/0969725X.2011.641344

[ref122] Rosseel Y. Oberski D. Byrnes J. Vanbrabant L. (2017). Package ‘lavaan’.

[ref123] Salas Reyes R. Nguyen V. M. Schott S. Berseth V. Hutchen J. Taylor J.. (2021). A research agenda for affective dimensions in climate change risk perception and risk communication. Front Climate 3, 1–10. doi: 10.3389/fclim.2021.751310

[ref124] Sanchez M. J. Lafuente R. (2010) Defining and measuring environmental consciousness. Rev. int. sociol. 68, 731–755. doi: 10.3989/ris.2008.11.03

[ref125] Schreiber J. B. Nora A. Stage F. K. Barlow E. A. King J. (2006). Reporting structural equation modeling and confirmatory factor analysis results: a review. J Educ Res 99, 323–338. doi: 10.3200/JOER.99.6.323-338

[ref126] Searle K. Gow K. (2010). Do concerns about climate change lead to distress? Int J Climate Change Strategies Manag 2, 362–379. doi: 10.1108/17568691011089891

[ref127] Seebauer S. Winkler C. (2020). Should I stay or should I go? Factors in household decisions for or against relocation from a flood risk area. Glob Environ Chang 60:102018. doi: 10.1016/j.gloenvcha.2019.102018

[ref128] Sheppes G. Suri G. Gross J. J. (2015). Emotion regulation and psychopathology. Annu Rev Clin Psychol 11, 379–405. doi: 10.1146/annurev-clinpsy-032814-112739 25581242

[ref129] Simione L. Gnagnarella C. (2023). Humor coping reduces the positive relationship between avoidance coping strategies and perceived stress: a moderation analysis. Behav Sci 13:179. doi: 10.3390/bs13020179, PMID: 36829408 PMC9952361

[ref130] Skurka C. Niederdeppe J. Romero-Canyas R. Acup D. (2018). Pathways of influence in emotional appeals: benefits and tradeoffs of using fear or humor to promote climate change-related intentions and risk perceptions. J Commun 68, 169–193. doi: 10.1093/joc/jqx008

[ref131] Smith N. Leiserowitz A. (2014). The role of emotion in global warming policy support and opposition. Risk Anal 34, 937–948. doi: 10.1111/risa.12140, PMID: 24219420 PMC4298023

[ref132] Snyder C. R. (2000). “Hypothesis: there is hope” in Handbook of hope. Editor. C.R. Snyder. (San Diego, California: Academic Press), 3–21.

[ref133] Stern P. C. (2000). New environmental theories: toward a coherent theory of environmentally significant behavior. J Soc Issues 56, 407–424. doi: 10.1111/0022-4537.00175

[ref134] Stokols D. Misra S. Runnerstrom M. G. Hipp J. A. (2009). Psychology in an age of ecological crisis: from personal angst to collective action. Am Psychol 64, 181–193. doi: 10.1037/a0014717, PMID: 19348519

[ref135] Stone B. (2012). The City and the coming climate. New York, NY: Cambridge University Press.

[ref136] Syropoulos S. Markowitz E. M. (2022). Perceived responsibility to address climate change consistently relates to increased pro-environmental attitudes, behaviors and policy support: evidence across 23 countries. J Environ Psychol 83:101868. doi: 10.1016/j.jenvp.2022.101868

[ref137] Tabachnick B. G. Fidell L. S. (2019). Using multivariate statistics. New York, NY: Pearson.

[ref138] Tamir M. (2016). Why do people regulate their emotions? A taxonomy of motives in emotion regulation. Personal Soc Psychol Rev 20, 199–222. doi: 10.1177/1088868315586325, PMID: 26015392

[ref139] Thompson S. C. G. Barton M. A. (1994). Ecocentric and anthropocentric attitudes toward the environment. J Environ Psychol 14, 149–157. doi: 10.1016/S0272-4944(05)80168-9

[ref140] Thornton P. K. Ericksen P. J. Herrero M. Challinor A. J. (2014). Climate variability and vulnerability to climate change: a review. Glob Chang Biol 20, 3313–3328. doi: 10.1111/gcb.12581, PMID: 24668802 PMC4258067

[ref141] Tomassini L. Lancia M. Gambelunghe A. Zahar A. Pini N. Gambelunghe C. (2024). Exploring the Nexus of climate change and substance abuse: a scoping review. Int J Environ Res Public Health 21:896. doi: 10.3390/ijerph21070896, PMID: 39063473 PMC11277026

[ref142] Uppalapati S. Ballew M. Campbell E. Maibach E. (2023). The prevalence of Climate Change Psychological Distress among American adults. Yale University and George Mason University. New Haven, CT: Yale Program on Climate Change Communication.

[ref143] Valquaresma A. Coimbra J. L. Costa P. (2022). Creative self-efficacy scale for children and adolescents (CASES): a development and validation study. Int J Psycholog Res 15, 55–69. doi: 10.21500/20112084.5410, PMID: 36199520 PMC9529283

[ref144] van der Linden S. (2014). On the relationship between personal experience, affect and risk perception: the case of climate change. Eur J Soc Psychol 44, 430–440. doi: 10.1002/ejsp.2008, PMID: 25678723 PMC4312984

[ref145] Verlie B. (2019). Bearing worlds: learning to live with climate change. Environ Educ Res 25, 751–766. doi: 10.1080/13504622.2019.1637823

[ref146] Verplanken B. Roy D. (2013). "my worries are rational, climate change is not": habitual ecological worrying is an adaptive response. PLoS One 8, 1–6. doi: 10.1371/journal.pone.0074708, PMID: 24023958 PMC3762778

[ref147] Watson D. Clark L. A. (1994). The PANAS-X: Manual for the positive and negative affect schedule-expanded form. Iowa City, Iowa: Psychology Publications, University of Iowa.

[ref148] Watson D. Clark L. A. Tellegen A. (1988). Development and validation of brief measures of positive and negative affect: the PANAS scales. J Pers Soc Psychol 54, 1063–1070. doi: 10.1037/0022-3514.54.6.1063 3397865

[ref149] White C. W. (2018). Re-envisioning hope: anthropogenic climate change, learned ignorance, and religious naturalism. J Religion Sci 53, 570–585. doi: 10.1111/zygo.12405

[ref150] Wong-Parodi G. Feygina I. (2021). Engaging people on climate change: the role of emotional responses. Environ Commun 15, 571–593. doi: 10.1080/17524032.2020.1871051, PMID: 40640861

[ref151] Zaremba D. Kulesza M. Herman A. M. Marczak M. Kossowski B. Budziszewska M.. (2022). A wise person plants a tree a day before the end of the world: coping with the emotional experience of climate change in Poland. Curr Psychol 42, 27167–27185. doi: 10.1007/s12144-022-03807-3, PMID: 36258889 PMC9561312

[ref152] Zummo L. Janney B. Sherman H. Whiting M. Giron J. (2025). Hope for the future: Strategic frames for learning within an innovative climate exhibit. US: American Educational Research Association.

