# Supplementary material for: From eco-consciousness to apathy: the ECO-SHADOW inventory to assess cognitive and behavioral affect regulation and its role in climate action
Source: Front Psychol. 2025 Sep 9;16:1575185. doi: 10.3389/fpsyg.2025.1575185 (PMC12454401; doi:10.3389/fpsyg.2025.1575185)
Supplement: Supplementary file 1 [file Supplementary_file_1.docx]

Supplementary Material

# Supplementary Appendix: Introduction to the survey

[Region]'s diverse geography, from mountains to deserts, brings many climate conditions. Climate change is a change in the usual weather, such as how much rain that place gets or the average temperature in a given season. Such changes to local climates have been reported all over the world. In [region], scientists have observed significant changes and impacts on [region]’s local climates, including:

 • Higher average temperatures during the summer months.
 • Major droughts reduced the overall precipitation and drove the shrinking [lakes].
 • Declines in wildlife, such as brine shrimp populations and migratory bird populations living [near the region].
 • Variance in the average snowfall impacting winter recreational sports.

 Please note: Thinking about the impacts of [region]’s changing climate may or may not be emotionally challenging. **The following survey aims to explore how people *typically respond to climate change* in their lives. There are lots of ways, so we are interested in your responses to climate change.**

 The following sections of this survey will present several possible ways. **We want to know to what extent you've been doing what the item says,** i.e., *how much or how frequently*. Don't answer based on whether it seems to be working or not—just whether or not you're doing it.

 Try to rate each item separately in your mind from the others. Make your answers as true for you as you can.

*******************************************************************************

**Additional notes to adapt the ECO-SHADOW measure for future research:**

1. To limit the time required, researchers can adopt one or a few specific scales (such as Eco-consciousness and Apathy) to capture constructs of interest without needing to administer all nine of the independent scales.
2. For items 4.01, 4.02, and 4.03, we suggest adding climate change context in these sentences. The suggested prefix that is being tested is “To deal with climate change,...”. For example, for item 4.01, the revised version should say, “To deal with climate change, I've been trying to find comfort in my religion or spiritual beliefs.”
3. For future use, researchers can edit the introduction that was utilized to adapt it to the region they are targeting (e.g., could be a state or country). Researchers could also add specific language to their introduction, specifically about facts on climate change that they present to all participants *before* they start the ECO-SHADOW inventory. Additional resources to add content can be found here: <https://www.ipcc.ch/report/ar6/wg1/downloads/report/IPCC_AR6_WGI_SPM.pdf> (international); <https://nca2023.globalchange.gov/> (national).

**2 The ECO-SHADOW (Eco-consciousness, Conflict, Outcast, Spirituality, Hope, Apathy, Doom, Overplay, and Withdrawal) inventory items.**

| # | Strategy descriptor | Item |  |  |  |  |
| --- | --- | --- | --- | --- | --- | --- |
| Factor 1: Eco-consciousness | | | | |  |  |
| 1.01 | Information seeking | I've been searching for information about what I as an individual can do. |  |  |  |  |
| 1.02 | Problem-focused-coping self | I've been thinking about what I myself can do. |  |  |  |  |
| 1.03 | Problem-focused-coping social | I've been talking with my family and friends about what one can do to help. |  |  |  |  |
| 1.04 | Planning | I've been thinking hard about what steps to take. |  |  |  |  |
| 1.05 | Eco-advocacy | I've been encouraging people around me to become more aware of environmental issues. |  |  |  |  |
| 1.06 | Active-coping | When considering the challenges of climate change, I feel it is important to look for things that I can address and change in my everyday life. | | | |  |
| 1.07 | Rumination | I've been thinking about the climate change issues again and again. |  |  |  |  |
| 1.08 | Eco-consciousness cognitive | Climate change has forced me to change the way I think about and view how we live in and use our natural environment in [state/region]. | | | |  |
| 1.09 | Sustainable-behavior | I've been trying to reduce my behaviors that contribute to climate change. | | | |  |
| 1.1 | Reflection | I've been continually thinking about what was bothering me, specifically about climate change. | | | |  |
| 1.11 | Connect with Environment | I always think about how my actions affect the environment. | | | |  |
| 1.12 | Significance | I've been thinking about all the different things in my life that climate change would impact. | | | |  |
| 1.13 | Moral-engagement | I feel a moral duty to do something about climate change. | | | |  |
| 1.14 | Volunteerism | I've been trying to connect with efforts that would help address climate change. | | | |  |
| 1.15 | Environmental-efficacy | I've been feeling that I can do something about climate change. | | | |  |
| 1.16 | Cognitive-reappraisal | I've been thinking of other ways to interpret the climate change problems. | | | |  |
| 1.17 | Concerned | It has been upsetting me that there seems to be so little that I can do to address environmental problems such as climate change. | | | |  |
| 1.18 | Change-of-habitat | I have seriously thought about alternative places to live because of the increasingly evident impacts of climate change. | | | |  |
| Factor 2: Conflict | | | | |  |  |
| 2.01 | Cognitive dissonance (behavioral) | When it comes to climate change, I feel that my thoughts and actions are contradictory. | | | | |
| 2.02 | Cognitive dissonance (cognitive) | I've been experiencing mental conflict because my climate change beliefs do not line up with my actions. | | | | |
| 2.03 | Ambivalence | Even though I believe in climate change, I don't feel that I am able to act sustainably and live an environmentally friendly life. | | | | |
| 2.04 | Wishful-thinking | I've been wishing I behaved more sustainably. | | | | |
| 2.05 | Procrastination | I've been putting off doing environmentally friendly behaviors that can be done. | | | | |
| 2.06 | Self-blame | I've been blaming myself for issues happening with climate change. | | | | |
| Factor 3: Outcast | | | | |  |  |
| 3.01 | Isolated | I've been feeling alone and isolated in my concerns about climate change. | | | | |
| 3.02 | Social exclusion | I've been feeling left out because of my beliefs about climate change. | | | | |
| 3.03 | Impairment | My concerns about climate change have been undermining my ability to get work done up to my potential. | | | | |
| 3.04 | Substance-abuse | I've been using alcohol or other drugs to make myself feel better about climate change problems. | | | | |
| 3.05 | Alienated | I've been engaging in environmental volunteerism. | | | | |
| 3.06 | Stonewalling | I've been refusing to have a discussion about climate change. | | | | |
| 3.07 | Hesitation | I've been hesitant to share my true feelings about climate change with others. | | | | |
| Factor 4: Spiritual-bodily practices | | | | |  |  |
| 4.01 | Religious belief | I've been trying to find comfort in my religion or spiritual beliefs. | | | | |
| 4.02 | Prayer-meditation | I've been praying or meditating. | | | |  |
| 4.03 | Relaxation | I've been trying to take deep breaths or exercise. | | | |  |
| Factor 5: Hope | | | | |  |  |
| 5.01 | Hope - Scientists | I have faith in scientists and people engaged in environmental organizations to come up with a solution in the future. | | | |  |
| 5.02 | Hope - Humanity | I have faith in humanity; I believe we together can do something about climate change. | | | |  |
| 5.03 | Hope – Solution | I've been thinking that the climate change problem will be solved in the future. | | | |  |
| 5.04 | Rational Optimism | I've been thinking that even though climate change is a big problem, one has to have hope. | | | |  |
| Factor 6: Apathy | | | | |  |  |
| 6.01 | Downplay | I've been thinking that the climate change threats have been exaggerated. | | | |  |
| 6.02 | Apathy | I do not care about climate change. | | | |  |
| 6.03 | Belittle | I've been thinking that the problem of depletion of natural resources is not as bad as many people make it out to be. | | | |  |
| 6.04 | Irrelevance | I can't be bothered to care about climate change. | | | |  |
| 6.05 | De-emphasizing | I feel that nothing serious will happen during my lifetime. | | | |  |
| 6.06 | Perspective-taking | I've been thinking that the climate change problem has not been too bad compared to other things. | | | |  |
| 6.07 | Indifference | I've been feeling indifferent to what is going on with climate change. | | | |  |
| 6.08 | Skepticism | I've been thinking that climate change is due to Earth's natural cycles, so humans can have little influence on it. | | | |  |
| 6.09 | Low-priority | I've been thinking that our society has many other challenges that need to be prioritized. | | | |  |
| 6.10 | Denial (emotional) | I say to myself, "This isn't real." | | | |  |
| Factor 7: Doom | | | | |  |  |
| 7.01 | Overwhelmed | I've been thinking that climate change problems seem too big for me to do anything about. | | | |  |
| 7.02 | Purposeless | I've been thinking that my efforts on climate change are too small to make a difference. | | | |  |
| 7.03 | Meaningless | I feel there is little meaning in the things I do, and it won't make a difference in addressing climate change. | | | |  |
| 7.04 | Limited | I've been thinking that it is not that I don't care, I just have a lot of other challenges to deal with. | | | |  |
| 7.05 | Powerless | I've been feeling helpless and I do not know how to overcome the climate change problem. | | | |  |
| 7.06 | Lost-cause | I think the environmental destruction is unavoidable and I don't think that things will get better. | | | |  |
| 7.07 | Other-blame | I've been feeling that the cause of climate change lies with others, such as policymakers, corporations,  people in power, and the media. | | | |  |
| Factor 8: Overplay | | | | |  |  |
| 8.01 | Humor | I've been making fun of the situation. | | | |  |
| 8.02 | Mocking | I've been making jokes about it. | | | |  |
| Factor 9: Withdraw | | | | |  |  |
| 9.01 | Experiential avoidance | I've been trying to avoid thinking about climate change-related feelings and problems. | | | |  |
| 9.02 | Worry (universal) | I've been worrying about myself, my relatives, future generations, animals, and nature | | | |  |
| 9.03 | Self-distraction | I've been turning to work or other activities to take my mind off issues related to climate change. | | | |  |
| 9.04 | Catastrophizing | I've been continually thinking how horrible the situation is. | | | |  |
| 9.05 | Information avoidance | I've been trying to avoid information about climate change issues. | | | |  |
| 9.06 | Nonacceptance | I've been unable to accept how bad the climate change problems have become. | | | |  |
| 9.07 | Suppression | I've been making an effort to hide my feelings. | | | |  |
| 9.08 | Behavioral Self-distraction | I've been doing something to think about climate change less, such as going to movies, watching TV, reading, daydreaming, sleeping, or shopping. | | | |  |

For scoring, an average of all items within each factor can be used to obtain a composite score for each of the nine scales.

**
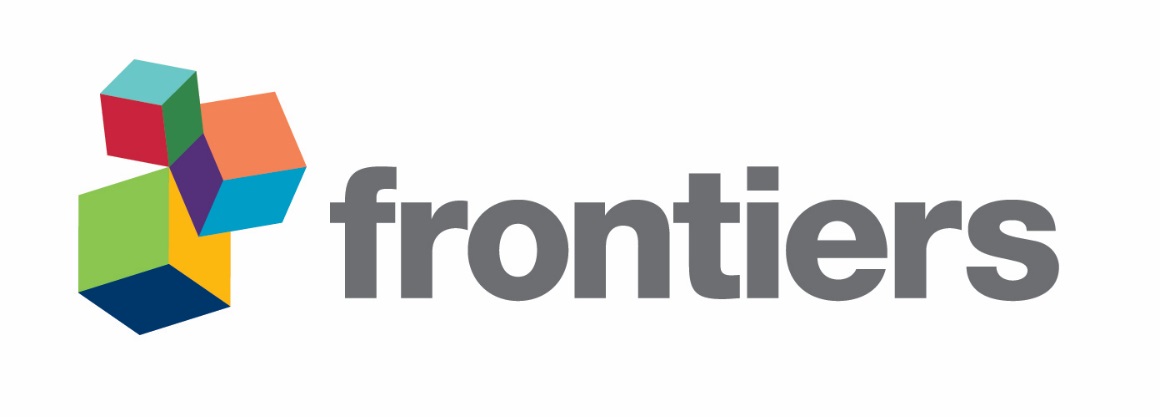
**
